# A gene-based score for the risk stratification of stage IA lung adenocarcinoma

**DOI:** 10.1186/s12931-023-02647-4

**Published:** 2024-01-04

**Authors:** Yanlu Xiong, Yongfu Ma, Kun Liu, Jie Lei, Jinbo Zhao, Jianfei Zhu, Wenchen Wang, Miaomiao Wen, Xuejiao Wang, Ying Sun, Yabo Zhao, Yong Han, Tao Jiang, Yang Liu

**Affiliations:** 1 Department of Thoracic Surgery, First Medical Center, Chinese PLA General Hospital and PLA Medical School, Beijing, China; 2 Department of Thoracic Surgery, Tangdu Hospital, Fourth Military Medical University, Xi’an, China; 3 Innovation Center for Advanced Medicine, Tangdu Hospital, Fourth Military Medical University, Xi’an, China; 4 Department of Epidemiology, Ministry of Education Key Laboratory of Hazard Assessment and Control in Special Operational Environment, School of Public Health, Air Force Medical University, Xi’an, China; 5 Department of Thoracic Surgery, Shaanxi Provincial People’s Hospital, Xi’an, China; 6 Department of Thoracic Surgery, Air Force Medical Center, Fourth Military Medical University, Beijing, China

**Keywords:** LUAD, Stage IA, Risk stratification, Prognosis, IA score, IAExpSuv

## Abstract

**Objective:**

We aim to molecularly stratify stage IA lung adenocarcinoma (LUAD) for precision medicine.

**Methods:**

Twelve multi-institution datasets (837 cases of IA) were used to classify the high- and low-risk types (based on survival status within 5 years), and the biological differences were compared. Then, a gene-based classifying score (IA score) was trained, tested and validated by several machine learning methods. Furthermore, we estimated the significance of the IA score in the prognostic assessment, chemotherapy prediction and risk stratification of stage IA LUAD. We also developed an R package for the clinical application. The SEER database (15708 IA samples) and TCGA Pan-Cancer (1881 stage I samples) database were used to verify clinical significance.

**Results:**

Compared with the low-risk group, the high-risk group of stage IA LUAD has obvious enrichment of the malignant pathway and more driver mutations and copy number variations. The effect of the IA score on the classification of high- and low-risk stage IA LUAD was much better than that of classical clinicopathological factors (training set: AUC = 0.9, validation set: AUC = 0.7). The IA score can significantly predict the prognosis of stage IA LUAD and has a prognostic effect for stage I pancancer. The IA score can effectively predict chemotherapy sensitivity and occult metastasis or invasion in stage IA LUAD. The R package IAExpSuv has a good risk probability prediction effect for both groups and single stages of IA LUAD.

**Conclusions:**

The IA score can effectively stratify the risk of stage IA LUAD, offering good assistance in precision medicine.

**Supplementary Information:**

The online version contains supplementary material available at 10.1186/s12931-023-02647-4.

## Introduction

With the increasing prevalence of low-dose spiral CT in lung cancer screening, the detection rate of stage IA lung adenocarcinoma (LUAD) characterized by small lung nodules is increasing, but the specific treatment for this disease still faces large challenges [1]. Standard treatment (lobectomy plus complete lymph node dissection without adjuvant therapy) has a good treatment effect for most stage IA LUAD patients but may also cause overtreatment for some low-risk populations and undertreatment for high-risk populations [2–5]. How to effectively classify the high- and low-risk subtypes of stage IA LUAD is an urgent problem that needs to be solved clinically. The risk classification of stage IA LUAD based on pathological morphology and radiological characteristics shows that these characteristics are good indicators [4–8]. However, screening for high-risk groups still remains insufficient, and the clinical application still faces doubts and inconveniences. The massive gene expression characteristics brought by the progress of omics technology can assist greatly in understanding carcinogenesis and the precise treatment of cancers [9]. In this study, we established a gene-based classifying score (IA score) and R package (IAExpSuv) for the risk stratification of stage IA LUAD that demonstrated good clinical application value.

## Methods

### Transcriptomic and clinical information

The RNASeq, DNA copy number, somatic mutation data and BiospecimenClinicalData of the TCGA were downloaded through the R package TCGA-assembler [10, 11]. Clinical information was acquired from the TCGA Pan-Cancer Clinical Data Resource (TCGA-CDR) [12]. The transcriptome data and clinical information of GSE13213, GSE26939, GS30219, GSE31210, GSE41217, GSE42127, GSE50081, GSE63459, GSE68465, GSE68571, and GSE72094 were all downloaded by the R package GEO Query [13]. The transcriptome data and clinical information of early-stage LUAD (carcinoma in situ, AIS; microinvasive adenocarcinoma, MIA; invasive adenocarcinoma, IAC) of our center have been deposited in the Genome Sequence Archive in National Genomics Data Center, China National Center for Bioinformation/Beijing Institute of Genomics, Chinese Academy of Sciences (GSA-Human: HRA005169). Clinical data in the SEER database were retrieved via SEER*Stat version 8.3.9.2 software for external validation [14]. Detailed information about Platform or Methods, Normalization, Histological type, Clinical stage and etc. is provided in Additional file 1: Table S1. Original data can be found in https://www.ncbi.nlm.nih.gov/geo/, https://portal.gdc.cancer.gov/, https://ngdc.cncb.ac.cn/gsa-human, and https://seer.cancer.gov/.

### Study design and bioinformatic analysis

**Fig. 1:**
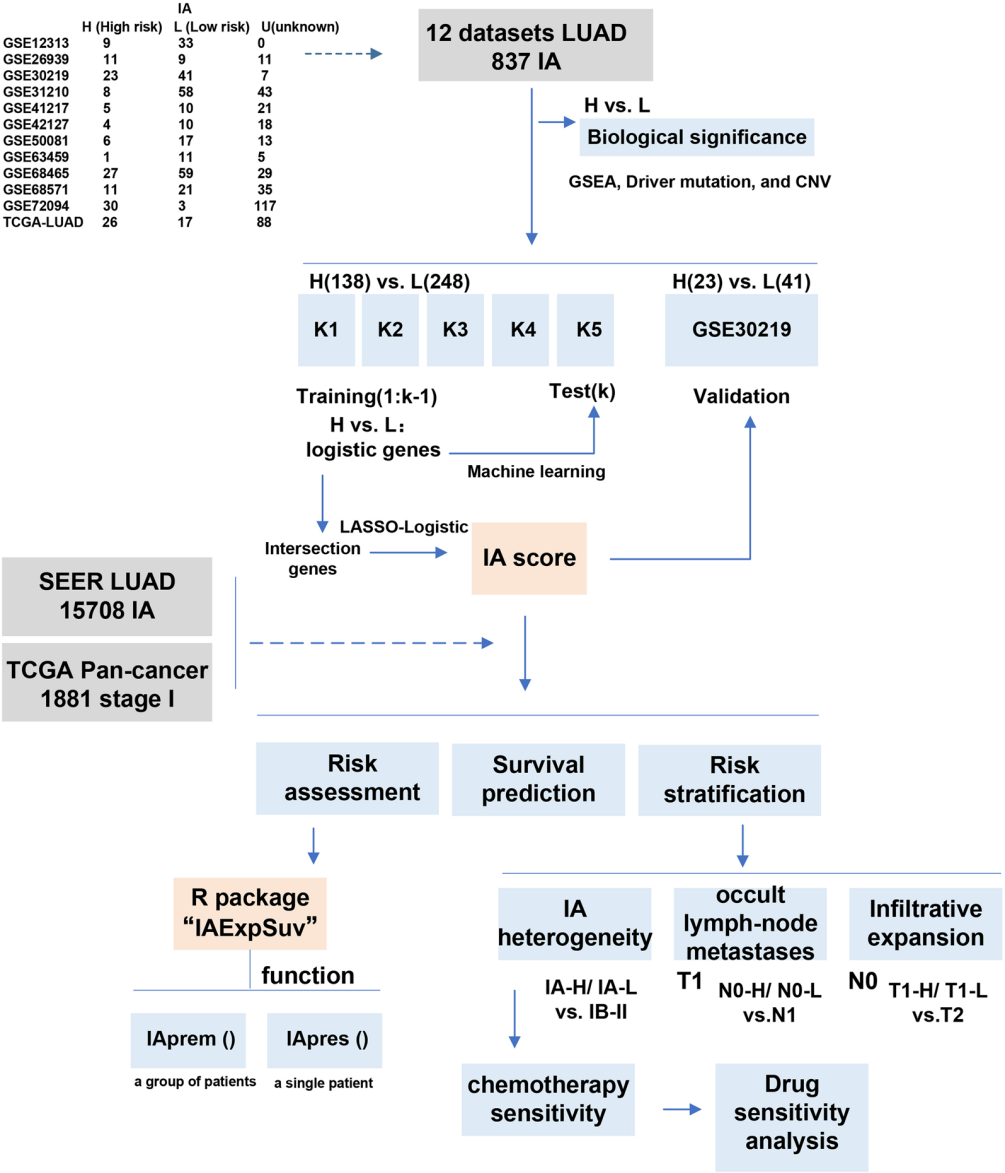
Study design. *TCGA* The Cancer Genome Atlas, *LUAD* lung adenocarcinoma, *SEER* Surveillance Epidemiology and End Results, *GSEA* Gene Set Enrichment Analysis, *CNV* copy number variation, *LASSO* least absolute shrinkage and selection operator

Our workflow is shown in Fig. 1. Since metastasis and recurrence of non-small-cell lung cancer usually occur within 5 years after treatment, patients who are still alive for more than 5 years are often regarded as clinically cured [15–17]. Therefore, we used the 5-year survival rate as the criterion for identifying high-risk or low-risk subtypes of stage IA LUAD. Then 12 datasets of 837 patients with stage IA LUAD were integrated and three canonical batch effect removal methods in gene expression matrix: z-scaling (Standardization), removeBatchEffect (limma package) and Combat (sva package) were used to normalize the transcriptome data for batch effect removal [18, 19]. UMAP (UMAP package) was used to evaluate the effect of batch removal for its excellent performance in reflecting the global structure of data and improving the operation speed [20, 21]. Next, the biological differences of high-risk and low-risk subtypes of stage IA LUAD were analyzed, where Gene set enrichment analysis (GSEA) was used to analyze different pathways (ClusterProfiler package) [22] and the TCGAmutations package and maftools package were used to analyze mutation status [23]. Then, the gene-based risk score (IA score) was established, tested and validated using k-fold cross validation by machine learning (K-nearest neighbor (knn), naive Bayes (nb), neural network (nnet), partial least squares (pls), support vector machine (svm), stochastic gradient boosting (gbm), boosting (C5.0), extreme gradient boosting (xgbTree), random forest (rf), bagged classification and regression tree (treebag) and stacking were used for machine learning of transcriptome data (caret, e1071 and h2o packages) [24, 25]). Least absolute shrinkage and selection operator (LASSO)—Logistic regression were used for dimension reduction, score establishment, risk factor analysis and probability prediction (glmnet package) [26]). Furthermore, the role of the IA score in prognostic assessment, chemotherapy prediction (The Connectivity Map database was used for drug sensitivity screening [27, 28]), identification of occult metastasis and invasion of stage IA LUAD was evaluated. Finally, we developed the R package IAExpSuv based on the IA score for risk probability prediction in groups or for single stages of IA LUAD, where the GitHub platform and devtools packages were used for R package development and storage. A total of 15,708 patients with stage IA LUAD from the SEER database were used to evaluate the classical clinical parameters, and 1881 patients with stage I cancers from the TCGA-pan database were used to evaluate the significance of this score for pancancer (The number of patient samples is based on the overall survival (OS) analysis statistics).

### Statistical analysis

Kaplan‒Meier curves were applied to display survival probability, the log-rank test was used for statistical analysis, and Cox analysis was used to identify the risk factors for survival (survival and survminer package). The Benjamini‒Hochberg (BH) method was used for p value correction for multigroup comparisons. Receiver operating characteristic (ROC) curves were used for diagnostic analysis (pROC package). Decision curve analysis was used to evaluate the clinical benefit (dcurves and rmda packages) [29]. The function relweights was applied to evaluate the relative importance of model variables.The Kruskal‒Wallis test (function: kruskal.test) was executed for differential gene analysis under the conditions of FDR < 0.05 and abs (log2(fold change [median])) > 0.3. Nomograms were used to visualize the probability prediction of the risk scores, and bootstrapping with 1000 resamples was used for testing (functions: nomogram and calibrate). The ggplot2 package was used for graphing. R language was adopted for the above operations, with a two-sided p value (or adj. p value) < 0.05 considered statistically significant [30].

## Results

### Biological differences

**Fig. 2:**
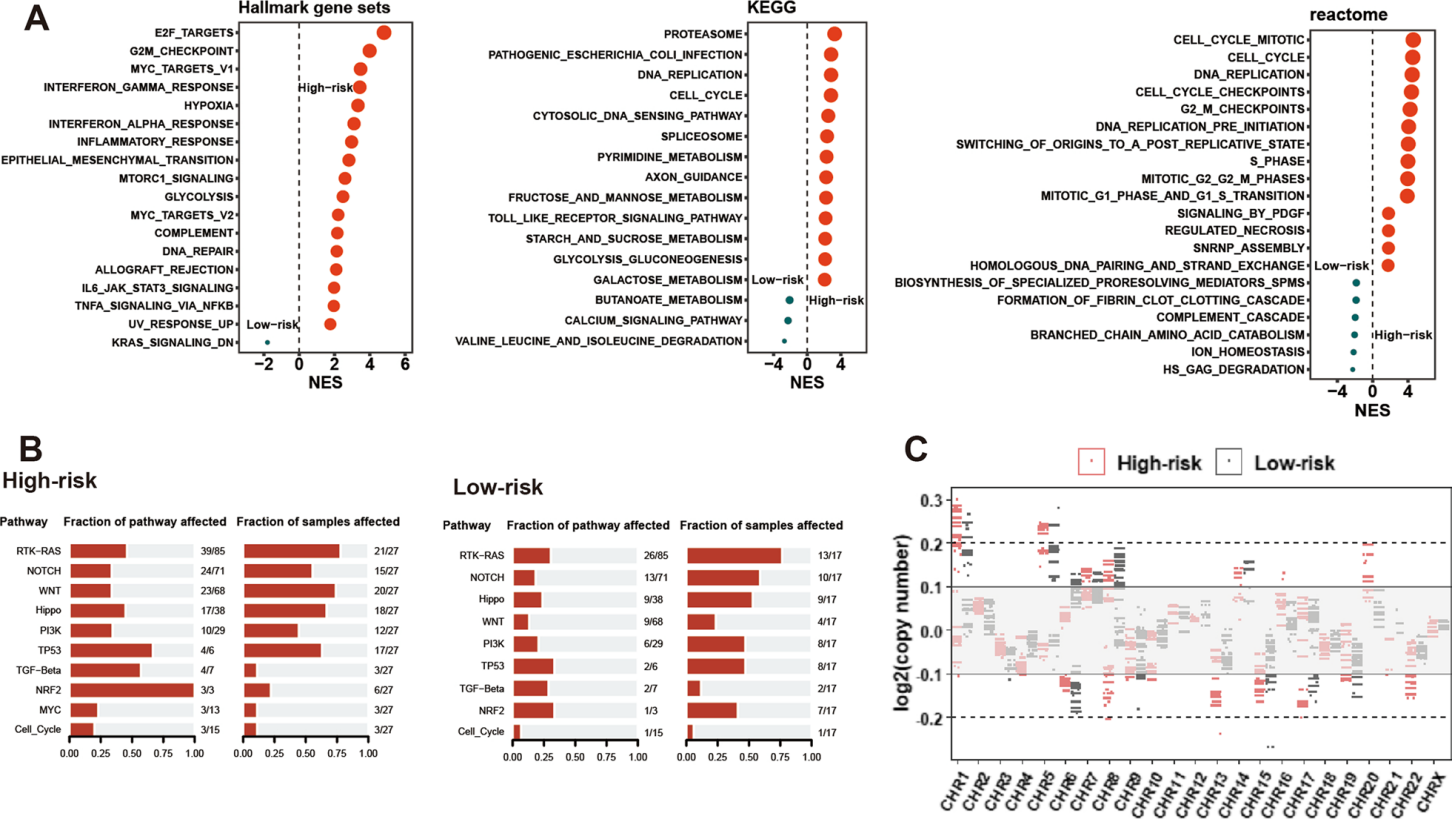
Biological differences in high- and low-risk stage IA lung adenocarcinoma. **A** Different biological pathway enrichment for high-risk and low-risk stage IA LUAD by GSEA in three database (Hallmark gene sets [left], KEGG [middle], and reactome [right]) for 12 datasets merging; **B** Canonical driver pathway mutations in high-risk (left) and low-risk (right) stage IA LUAD in TCGA-LUAD dataset; **C** Copy number variations in high-risk and low-risk stage IA LUAD in TCGA-LUAD dataset. *LUAD* lung adenocarcinoma, *GSEA* Gene Set Enrichment Analysis, *TCGA* The Cancer Genome Atlas

We divided 837 patients with stage IA LUAD in 12 datasets into a high-risk group (H) (161 patients who died within 5 years), a low-risk group (L) (289 patients who survived more than 5 years), and an unknown group (U) (387 patients with an unknown 5-year survival status) (Fig. 1). We first analyzed the biological differences between H and L. The batch effect refers to the nonbiological, technological or experimental variation introduced in the experimental process, which often affects the combined processing of multiple omics datasets. The selection of the batch effect removal method mainly considers different data characteristics, eliminates technological and experimental variation, and prevents excessive adjustment of data, which could hide real biological variations and signals present in the original data [18, 31]. We selected three batch effect removal methods commonly used in gene expression profiling: z-scaling (Standardization), removeBatchEffect (R package: limma) and Combat (R package: sva). We found that z-scaling performs well in removing batch effects and preserving inherent biological differences, while the other two methods are less effective (Additional file 8: Figure S1). We ranked genes by fold change of median (H vs. L) and performed GSEA. We found that H showed more enrichment of malignant pathways (hallmark gene sets, KEGG, reactome) (Fig. 2A). The TCGA-LUAD dataset possessed data on driver mutation and copy number status, and in this dataset, we found that H had more cancer driver mutations (Fig. 2B) and showed more copy number variations (CNVs) than L (Fig. 2C).

### Machine learning to establish the risk score

**Fig. 3:**
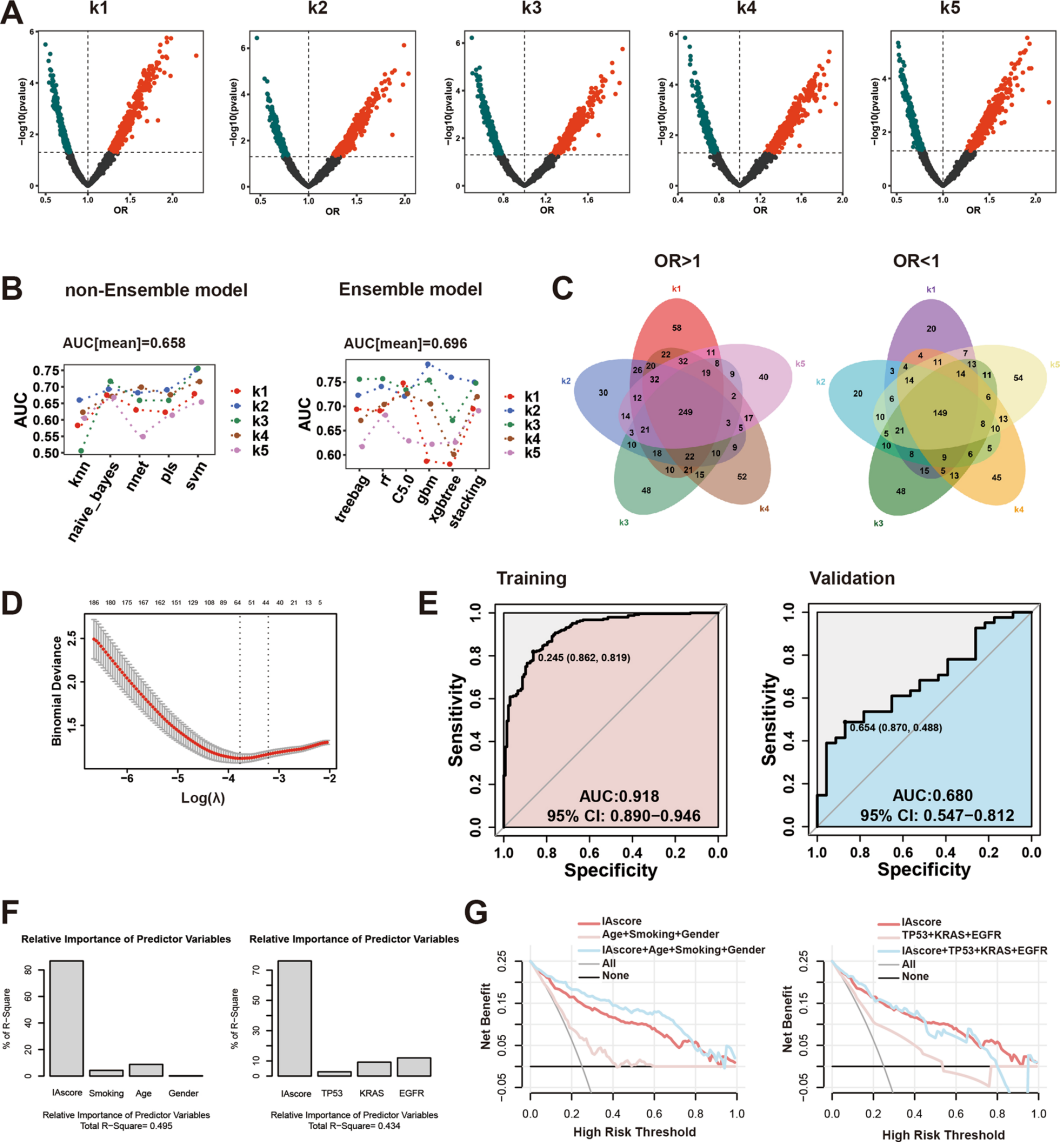
Machine learning to determine risk score for discriminating high-and low-risk stage IA lung adenocarcinoma. **A** Risk-related genes by logistic regression (high-risk, H vs. low-risk, L) in each training set (k[1: k-1]; k = 1,2,3,4,5); **B** Prediction effect (classifying H and L) of risk-related gene (1: k-1) in test set (k) by five machine learning algorithms (left) and six ensemble models (right); **C** Intersection of risk-related genes in five trials: OR > 1 (left) and OR < 1 (right); **D** LASSO regression to filtrate intersection genes for establish risk score (IA score) for 386 samples (11 datasets not including GSE30219); **E** Prediction effect of IA score (classifying H and L) in training set (11 datasets not including GSE30219) (left) and external validation set (GSE30219) (right); **F** Relative weight of IA score and classical demographic factors (left) and driver mutations (right) for risk classifying; **G** Discrimination power of IA score and classical demographic factors (left) and driver mutations (right) for risk classifying in decision curve analysis. *OR* Odds Ratio, *LASSO* least absolute shrinkage and selection operator

We applied GSE30219, which had relatively more samples (both H and L), as the external validation set and the remaining 386 patients (11 datasets) as the machine learning population (Fig. 1). We first adopted k-fold cross validation (k = 5). We divided the 386 samples into five parts and identified the risk-related genes in the training set (1: k-1) by logistic regression (H vs. L) (Fig. 3A). We conducted a sanity check of general information, including survival status (high-risk vs. low-risk), sex ratio (female vs. male), age: median (min, max) and smoking status (yes vs. no) (notably, some patients had unclear smoking history) in 5 different populations (Additional file 2: Table S2). We found that the distribution of characteristics in the 5 populations was almost evenly balanced, and the classification was reasonable. The prediction effect of corresponding risk-related genes (1: k-1) in the test set (k) was evaluated by five machine learning algorithms (non-ensemble model) and six ensemble models (Fig. 1). The area under the curve (AUC) was used to assess the prediction effect. We found that the average prediction in the test set was 0.7 (0.658 for non-ensemble algorithms and 0.696 for ensemble algorithms), mainly because of the inherent limitation of the data (small sample size of the high-risk IA type) (Fig. 3B). We took the intersection of the risk-related genes (398) in the five trials (Fig. 3C) and further filtered genes by LASSO regression and finally established the risk score (IA score) based on 64 genes in the above 386 samples by LASSO-Logistic regression (Fig. 3D). The prediction performance of the IA score in the training set (11 datasets not including GSE30219) reached 0.9 and reached the upper limit of the mean prediction (0.7) in the external validation set (GSE30219) (Fig. 3E). Compared with clinicopathological factors, the IA score showed far superior discrimination power via relative weight analysis and decision curve analysis (12 datasets) (Fig. 3F, G). Detailed information on the constituent genes related to the IA score, their biological significance, population distribution and even the detailed calculation formula of the IA score are shown in Additional file 3: Table S3, Additional file 4: Table S4, Additional file 9: Figure S2, Additional file 12: Supplementary Text. We also set up a scoring system to identify high and low risk subgroup in the stage IA patients based on feature screening of gene clusters and compared it with the IA score model; detailed information on this model is also provided in Additional file 5: Table S5, Additional file 10: Figure S3, Additional file 12: Supplementary Text.

### Survival prediction

**Fig. 4:**
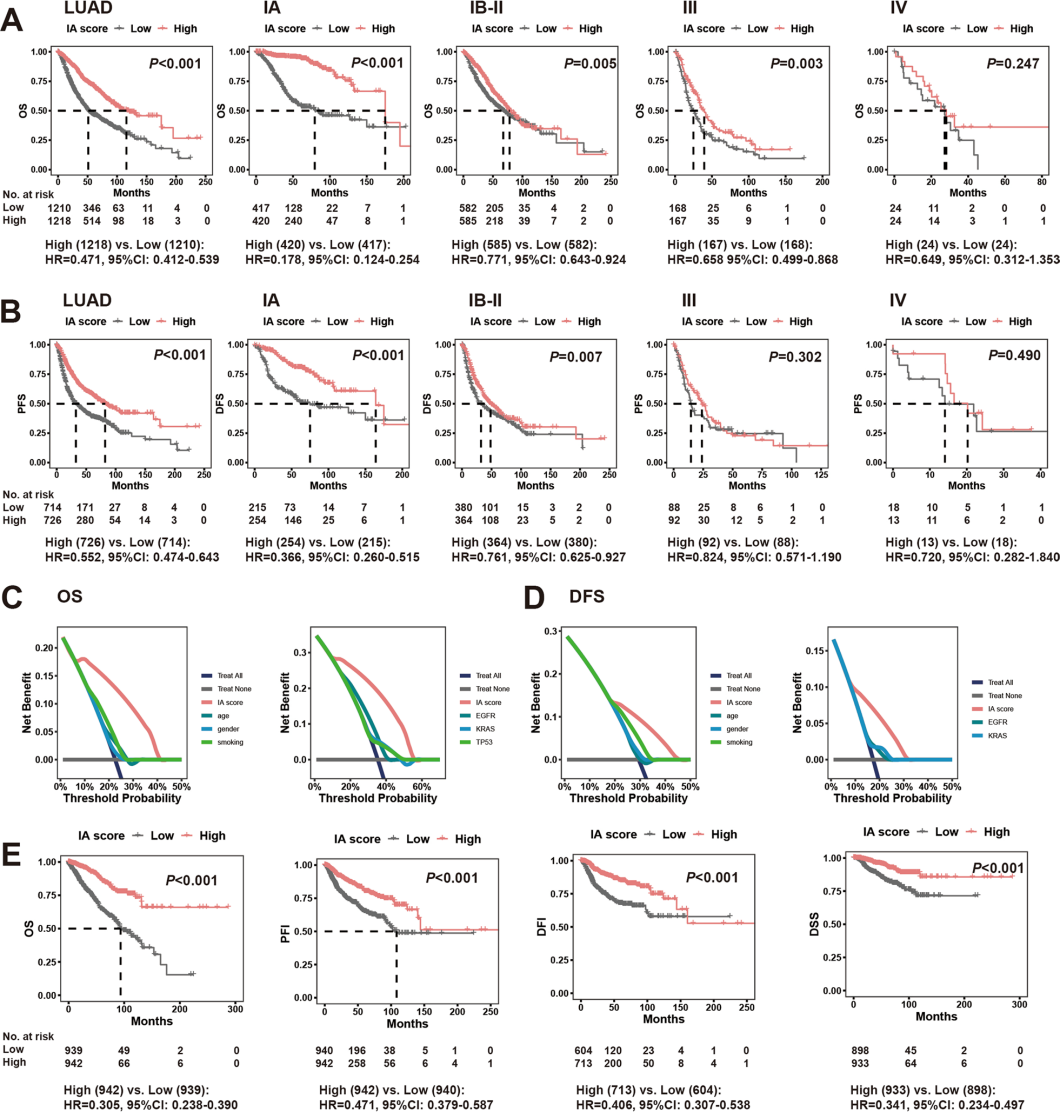
Prognostic effect of IA score in stage IA lung adenocarcinoma. OS (**A**) and DFS/PFS (**B**) curves for two categories of patients for whole LUAD and its different stages (IA, IB-II, III, and IV) according to the value of IA score (median value as the cutoff); Efficacies of IA score and classical demographic factors (left) and driver mutations (right) for predicting OS (**C**) and DFS (**D**) in decision curve analysis; **E** survival curves for two categories of patients for stage I cancers according to the value of IA score (median value as the cutoff). *OS* overall survival, *DFS* disease-free survival, *PFS* progression-free survival, *LUAD* lung adenocarcinoma, *PFI* progression-free interval, *DFI* disease-free interval, *DSS* disease-specific survival

Taking the median IA score as the cut off, we evaluated the prognostic effect of the IA score for LUAD. We found that the IA score could significantly predict the OS and disease-free survival (DFS)/progression-free survival (PFS) of overall LUAD; subgroup analysis found that the IA score could clearly predict the prognosis in stage IA and IB-II patients, and the prediction effect for stage IA was better than that for stage IB-II (OS, HR[IA]: 0.178 vs. HR[IB-II]: 0.771; DFS, HR[IA]: 0.366 vs. HR[IB-II]: 0.761) (Fig. 4A, B). However, the prediction effect for stage III and IV patients was not good (Fig. 4A, B). We combined the IA score with common classical demographic factors and driver mutations to identify independent prognostic factors for stage IA LUAD. We found that advanced age, male sex, smoking history, TP53 Mut, KRAS Mut, EGFR wild-type, and IA score were factors influencing OS in univariate Cox analysis, but only IA score, male sex, and TP53 Mut were independent factors influencing OS in multivariate Cox analysis (Additional file 6: Table S6). For DFS, advanced age, smoking history, EGFR wild-type, and IA score were influencing factors in univariate Cox analysis, but only IA score was an independent influencing factor in multivariate Cox analysis (Additional file 7: Table S7). Furthermore, we demonstrated that the prediction effect of the IA score on survival was far better than that of classical pathological factors and driver mutations by decision curve analysis (Fig. 4C, D). In the TCGA-pan cancer database, we found that the IA score still had a satisfactory predictive effect for stage I cancers (Fig. 4E).

### Risk stratification

**Fig. 5:**
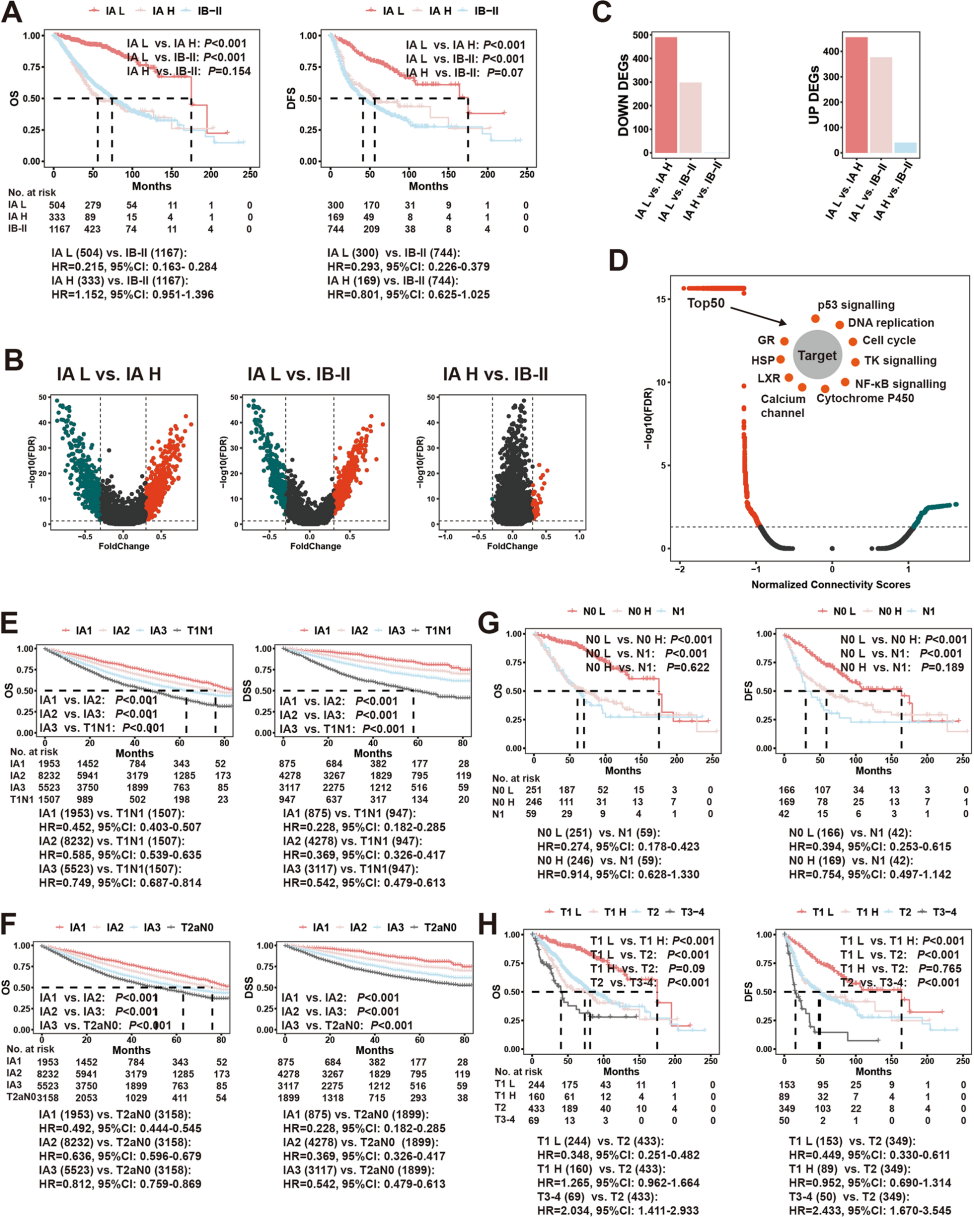
Significance of IA score in risk stratification of stage IA lung adenocarcinoma. **A** OS (left) and DFS (right) curves between two risk types of stage IA LUAD patients divided by median of IA score (IA H and IA L; IA score is a prognostic protective score) and stage IB-II LUAD patients; **B** volcano plots for DEGs between IA L and IA H (left), between IA L and IB-II (middle), and between IA-H and IB-II (right); **C** Down (left) and Up (right) DEGs between IA L and IA H, between IA L and IB-II, and between IA-H and IB-II; **D** similarities between query signature (IA H vs. IA L) and touchstone signatures (compounds inhibiting certain pathways in lung cancer cell lines) in Connective Map database; **E** OS (left) and DSS (right) curves among IA1, IA2, IA3 and T1N1 LUAD patients in SEER database; **F** OS (left) and DSS (right) curves among IA1, IA2, IA3 and T2aN0 LUAD patients in SEER database; **G** OS (left) and DFS (right) curves between two risk types of stage IA LUAD patients divided by median of IA score (N0 H and N0 L) and stage T1N1 LUAD patients; **H** OS (left) and DFS (right) curves between two risk types of stage IA LUAD patients divided by median of IA score (T1 H and T1 L) and stage T2, T3–4 LUAD patients. *LUAD* lung adenocarcinoma, *OS* overall survival, *DFS* disease-free survival, *DSS* disease-specific survival, *DEGs* differentially expressed genes, *SEER* Surveillance Epidemiology and End Results

An important purpose of risk stratification for stage IA LUAD is to identify high-risk subgroups that may require more aggressive treatment, such as postoperative adjuvant chemotherapy. We first analyzed the similarity of prognosis and gene expression between known chemotherapy-sensitive groups (high-risk stage IB and stage II populations) and stage IA subgroups classified by IA score and classical staging groups to evaluate the efficacy. We found that although subtypes of stage IA LUAD in the 8th TNM staging (IA1/2/3) have different prognoses, the high-risk subtype (IA3) still had superior survival compared to stage IB-II disease (Additional file 11: Figure S4A). However, high-risk IA subtypes based on IA score (IA H, lower IA score) demonstrated similar prognosis with stage IB-II cancer, while IA H and IB-II diseases showed poorer prognosis than the low-risk IA subtype based on IA score (IA L, higher IA score) (Fig. 5A). Furthermore, differential gene analysis showed that IA H and IB-II disease had almost the same gene expression characteristics, while IA H and IB-II tumors showed large discrepancies in their gene expression profiles compared to IA L (Fig. 5B, C).

Furthermore, we explored whether people in the special group showed differences in chemotherapy sensitivity by comparing prognosis between the chemotherapy group and the nonchemotherapy group, thus directly evaluating the relationship between classical classification group, IA score classification group and chemotherapy sensitivity. We found that chemotherapy had a significant survival disadvantage for stage IA LUAD, even for high-risk subtypes (IA3) in the 8th TNM staging (Additional file 11: Figure S4B–D); while this survival disadvantage was not seen for high-risk subtypes classified by IA score (IA H), it was observed for low-risk subtypes classified by IA score (IA L) (Additional file 11: Figure S4E, F). Because of the small number of IA patients receiving chemotherapy, we further evaluated whether the IA score could indicate chemotherapy sensitivity for early LUAD (IB-II population). Similarly, chemotherapy had a survival disadvantage for the unscreened IB-II population; the survival disadvantage of chemotherapy was not seen for the IB-II high-risk population classified by IA score (IB-II H) but was presented for low-risk subtypes classified by IA score (IB-II L) (Additional file 11: Figure S4G–J).

Next, based on differential gene expression analysis of the high- and low-risk IA populations, we used the Connectivity Map database (a database that enables us to predict drugs whose related genes are highly correlated with diseases and their possible molecular mechanisms of action by comparing gene expression profile data to establish the association between genes, diseases and drugs [27, 28]) to find chemotherapeutic drugs that may be effective for high-risk IA populations. Based on the analysis results, we further demonstrated that the high-risk IA subgroup was sensitive to drugs related to classical tumor driver pathways, such as the p53 pathway and receptor tyrosine kinase (RTK) pathway (Fig. 5D).

A possible explanation for the presence of a high-risk subtype of stage IA LUAD is that the tumor has metastases or invasion that are more extensive than current modalities can detect (occult metastasis or invasion), i.e., there is an “underestimation” of both N and T status with current TNM staging [2]. We found that the high-risk subtype according to the 8th TNM staging (IA3) had a more significant survival advantage than both T1N1 and T2aN0; that is, the classification of the high-risk subtype of IA in the 8th TNM staging did not overlap with the existing TNM staging in the SEER database (Fig. 5E, F). We found that the survival of the high-risk subtype of stage IA LUAD classified by IA score (N0 H) was consistent with that of T1N1, reflecting the possible discriminative function of this IA score for occult lymph node metastasis (Fig. 5G). Similarly, the high-risk subtype of stage IA LUAD classified by IA score (T1 H) had consistent survival with T2N0. This strongly suggests the possible role of the IA score in discriminating occult invasion (the actual extent of tumor invasion beyond the T1 scope defined in current detection methods) (Fig. 5H).

### R package IAExpSuv

**Fig. 6:**
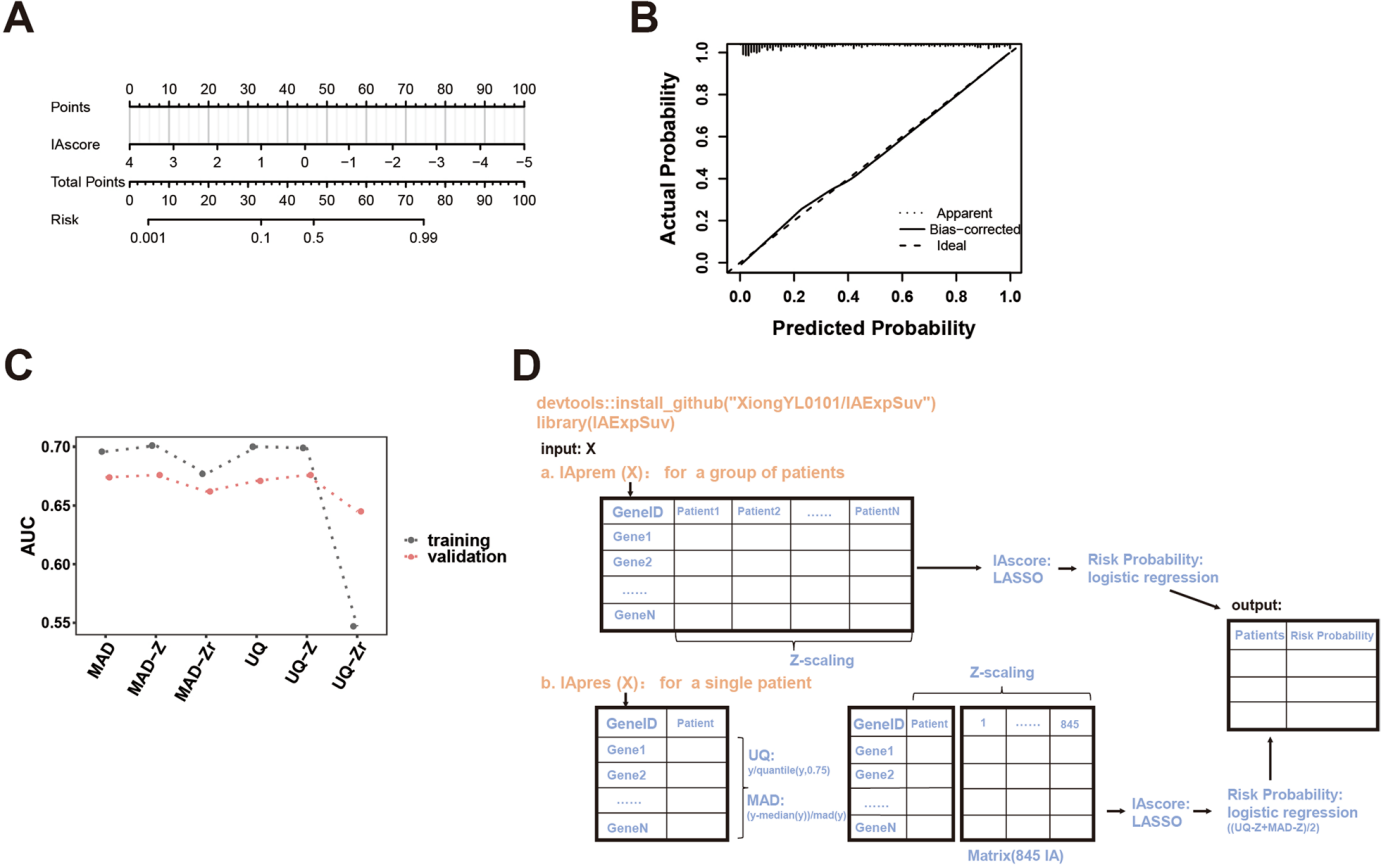
Establishing R package (IAExpSuv) for predicting survival risk probability. **A** A nomogram for predicting survival risk (five years) probability by logistic analysis (risk probability could be easily estimated by projecting total score calculated by IA score value to the lower point scale); **B** the calibration curves for the nomogram (Ideal prediction: 45-degree diagonal lines; Apparent prediction: entail cohorts; Bias-corrected prediction: bootstrapping for 1000 repetitions); **C** predicting efficacies (AUC) of IA score for an individual patient calculated by different procedures; **D** basic principle and download method for the R package IAExpSuv (Functions: IAprem and IApres) to predict the survival risk of stage IA LUAD patients. *AUC* area under the curve, *LUAD* lung adenocarcinoma

**Fig. 7:**
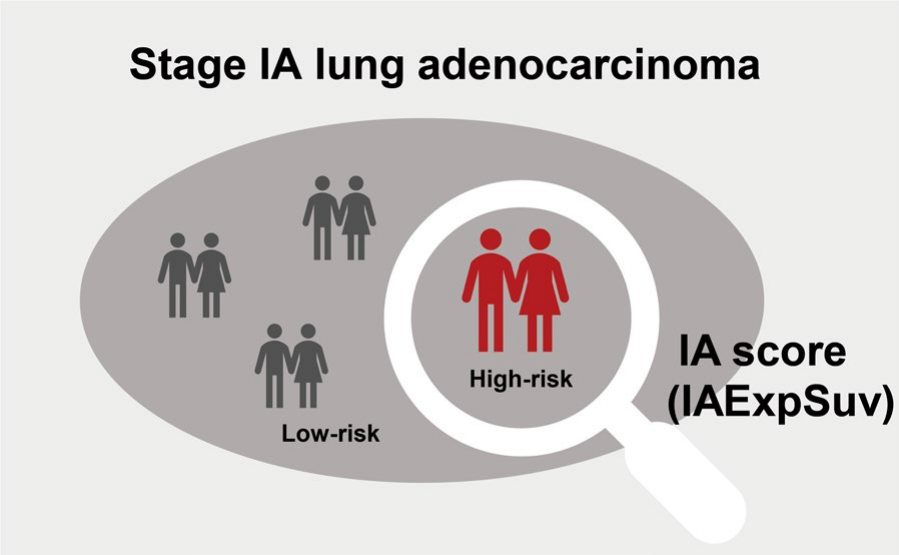
Central Picture. The IA score and R package (IAExpSuv) can effectively stratify the risk of stage IA LUAD

Through the logistic model, we established the survival risk model (death within 5 years) of stage IA LUAD based on the IA score, and the prediction efficiency of the model was good in cross-validation by bootstrapping with 1000 resamples (Fig. 6A, B). Since the IA score is based on a subset of Z-transformed samples, we explored how effective the risk prediction was for an individual patient. We first standardized the gene expression profiles of individual patients in two ways (MAD [median absolute deviation]: (y-median(y))/MAD (y); UQ [upper quartile]: (y)/quantile (y, 0.75); y, gene expression value) and then calculated the IA score and evaluated its risk prediction effect. After Z-transformation of MAD/UQ-standardized patients from the built-in dataset (845 IA patients with Z-transformation [MAD/UQ-Z] and without Z-transformation [MAD/UQ-Zr]), the IA score was calculated, and its risk prediction effect was evaluated. It was found that MAD/UQ-Z had a better prediction performance (AUC: 0.7, also reached the upper limit of the mean prediction in Figs. 3B, 6C. Hereby, we developed the R package IAExpSuv to predict the survival risk of IA patients, with the package including two functions, where function IAprem was for a group of patients (Z transformation, IA score transformed, and the logistic probability calculated), and function IApres was for the risk prediction of a single patient (self-normalization, Z-transformation with the built-in dataset, IA score transformed and logistic probability calculated) (Figs. 6D, 7).

## Discussion

Stage IA LUAD is a clinically early malignancy (T ≤ 3 cm N0 M0) and always possesses a favorable prognosis [32]. The LCSG821 study established anatomical lobectomy as the standard treatment for stage IA LUAD [33, 34]. Many clinical trials have proven that stage IA LUAD does not benefit from postoperative adjuvant chemotherapy [35, 36]. Therefore, the National Comprehensive Cancer Network (NCCN) guidelines recommend lobectomy plus systemic lymph node dissection without postoperative adjuvant chemotherapy as the standard of care for stage IA LUAD [32]. However, stage IA LUAD still has biological and prognostic heterogeneity, and its appropriate therapeutic strategy and postoperative management remain uncertain. Previous studies predicted the prognosis and optimized treatment of stage IA LUAD based on tumor size, radiological characteristics and pathological features. For example, the death risk increases with the tumor diameter for early LUAD [37]. The prognosis of stage IA LUAD containing ground-glass components is far better than that of pure solid tumors [5]. JCOG0804 confirmed that stage IA LUAD with T ≤ 2 cm and a consolidation tumor ratio (CTR) ≤ 0.25 could benefit from sublobular treatment (mainly wedge shaped), while JCOG0802 proved that segmental resection was better for stage IA LUAD with T ≤ 2 cm and a CTR > 0.5 [3, 6]. Stage I LUAD with lepidic growth has an excellent prognosis, while micropapilla, solid components, lymphovascular invasion, and spread through air spaces often predict a poor prognosis and benefits from adjuvant chemotherapy [4, 7, 8, 38]. Therefore, in the 2021 WHO histological classification, carcinoma in situ (AIS) with pure lepidic growth and lesions less than 3 cm were defined as precursor lesions of cancer, and LUAD with mainly lepidic growth, and lesions less than 3 cm with the presence of invasive components less than 5 mm were defined as microinvasion (T1mi) [32, 39]. Furthermore, invasive stage IA LUAD (invasive component > 5 mm) was further subdivided into T1a (< 1 cm), T1b (1 cm–2 cm), and T1c (2 cm–3 cm) in the 8th TNM staging [32].

However, the previous classification is still controversial. First, we found that the prognosis of the higher risk type (IA3) defined in the 8th TNM staging was still better than that of stage IB-II disease; thus, this classification cannot suggest directly whether to chemotherapy is needed. In addition, although existing studies believe that the maximum diameter of solid components can better distinguish prognosis than the maximum diameter of whole nodules, some studies believe that lung nodules containing ground-glass components belong to a special type of lung cancer and that size, proportion of solid components and maximum diameter of nodules cannot be used as prognostic risk factors [40–42]. Furthermore, it is difficult and subjective to distinguish histological morphology and pathological types. Moreover, there are often mixed pathological patterns with large variation, and the relationship of the proportion of different histological components and the extent of invasion with prognosis is still controversial. Therefore, there are still certain flaws in the current risk stratification of stage IA LUAD that thus limit precise clinical treatment.

We stratified the risk of stage IA LUAD from the perspective of gene expression characteristics. The IA score and application R package (IAExpSuv) have the following three advantages. First, this classification is more intuitive. The fundamental reason for the prognostic heterogeneity of stage IA LUAD is the heterogeneity of biological characteristics, and the gene expression characteristics largely dictate biological behavior. Therefore, our risk classification based directly on the differential gene expression characteristics of high- and low-risk populations is more thorough. The IA score can classify a high-risk type with a similar prognosis to that of stage IB-II disease, indicating that the IA score can predict stage improvement. The prognosis of the subgroup with high-risk stage IA LUAD (T1N0M0) according to IA score and of T1N1 disease (T1 stage disease with lymph node metastasis) was similar, suggesting that these patients may have occult lymph node metastasis. The prognosis of the subgroup with high-risk stage IA LUAD (T1N0M0) according to IA score and of T2N0 disease (T2 disease without lymph node metastasis) was similar, suggesting that tumor invasion and expansion in these patients may exceed that typically indicated by the T1 category. These findings suggest that the IA score can identify occult metastases and invasion (lymph node metastases and invasion not detected by current detection methods, i.e., underestimated N and T stages). High-risk stage IB and stage II patients can benefit from postoperative adjuvant therapy [43, 44]. Patients with N1 lymph node metastasis or T2 stage typically benefit from adjuvant chemotherapy [35, 45–47]. Therefore, it is reasonable to speculate that high-risk subtypes of stage IA LUAD classified by IA score present more aggressive biological characteristics and a poorer prognosis, indicating that these patients can benefit from adjuvant chemotherapy. We also further found that adjuvant chemotherapy is harmful for low-risk subgroups of stage IA LUAD patients classified by IA score, and this conclusion can also be extended to stage IB-II, suggesting that IA score can be used as an index for the classification of low-risk subtypes of early-stage LUAD. In addition, we found that for low-risk subtypes of stage IA LUAD patients classified by IA-score, the median survival time was 175 months for OS, with a five-year OS rate of 0.917, and the median DFS time was 175 months, with a five-year DFS rate of 0.789. The classic clinical trial with OS as the main endpoint (JCOG0802) indicated that low-risk subtypes of stage IA LUAD (T ≤ 2 cm and CTR > 0.5) benefiting from sublobectomy had a 5-year OS rate of 0.943, and another canonical clinical trial with DFS as the main endpoint (CALGB140503) indicated that low-risk subtypes of stage IA LUAD (T ≤ 2 cm) benefiting from sublobectomy had a 5-year DFS rate of 0.636 [3, 48]. The low-risk subtype of stage IA LUAD classified by IA score is similar to the existing low-risk subtype of stage IA LUAD that benefits from sublobectomy in terms of both OS and DFS. Therefore, the low-risk subtype of stage IA LUAD classified by IA score may benefit from sublobectomy. Second, we used 12 different datasets for training, testing and validation of the scoring system, so the IA score had good universality. Third, gene expression profiling only requires a small sample size, and the IA score calculation is intuitive and simple, so its clinical application would be acceptable.

In addition, we further analyzed the biological significance of the IA score and its possible therapeutic targets. Through gene enrichment analysis, we found that the 64 genes in the IA score model are enriched in signaling pathways such as proliferation and immunity, and malignant proliferation and immune microenvironment are key links in the malignant progression of early-stage LUAD [49–54], reflecting the biological rationality of IA score. We further screened key genes by analyzing the expression of these genes in early-stage LUAD tissues. We found that 13 genes showed consistency between risk classification and the expression distribution of AIS-MIA-IAC (eight genes, OR > 1, risk genes or oncogenes, sustained upward trend; five, OR < 1, protective gene or tumor suppressor gene, continuous downward trend). Most of these genes also play an important role in the evaluation of clinical features and malignant biological behavior of lung cancer. The details regarding the biological significance of these genes are shown in Additional file 12: Supplementary Text.

This study has some limitations. First, due to the sample size and quality of the public datasets (it is difficult to collect complete prognostic data of stage IA LUAD), as well as the inherent difficulties (patients with stage IA LUAD have a better prognosis, and it is difficult to identify those who died within five years), the efficacy of the IA score in the validation set could only reach 0.7. Additionally, owing to the lack of detailed data on chemotherapy (regimens and performance status of patients) and the small proportion of high-risk groups (sensitivity to chemotherapy) for early-stage LUAD (IA or IB-II), we could not directly identify IA patients who would benefit from chemotherapy through IA score, as we could only clearly and directly determine patients who should not receive chemotherapy. In addition, joint analysis of radiologic characteristics, pathological features and gene expression features cannot be achieved with public datasets. Additionally, the transition of the IA score from histological findings to blood test results remains to be verified.

## Conclusions

In summary, based on the biological differences between high-risk and low-risk patients with stage IA LUAD, we established the IA score and its R package IAExpSuv, which can be effectively used for prognostic classification and risk stratification to help provide precise treatment for stage IA LUAD.

### Supplementary Information

**Additional file 1: Table S1.** Details of the public datasets in this study.

**Additional file 2: Table S2.** General information for fivefold cross-validation of stage IA in training set.

**Additional file 3: Table S3.** Gene symbols and gene ids for 398 risk-related genes.

**Additional file 4: Table S4.** Gene symbols and gene ids for 64 LASSO screened genes.

**Additional file 5: Table S5.** Gene symbols and gene ids for 114 genes by LASSO within gene clusters.

**Additional file 6: Table S6.** Risk factors for overall survival in stage IA lung adenocarcinoma.

**Additional file 7: Table S7.** Risk factors for disease-free survival in stage IA lung adenocarcinoma.

**Additional file 8: Figure S1.** Analysis of the effect of batch removal of 12 datasets. Usage of U-MAP to exhibit distribution of total samples (A) or LUAD (B) in 12 datasets calculated by gene expression profiles without Z-scaling for normalization; Usage of U-MAP to exhibit distribution of total samples (C) or LUAD (D) in 12 datasets calculated by gene expression profiles with Z-scaling for normalization; Usage of U-MAP to exhibit distribution of different histological sample in total 12 datasets (E) and specific single datasets (F, TCGA-LUAD; G, GSE31210, H, GSE68465; I, GSE30219; J, GSE42127) by gene expression profiles with Z-scaling for normalization. Usage of U-MAP to exhibit distribution of total samples in 12 datasets calculated by gene expression profiles by removeBatchEffect (K) and Combat (L) methods for batch effect removal. LUAD, lung adenocarcinoma; AD, adenocarcinoma; SQC, squamous cell carcinoma; Normal, normal tissue

**Additional file 9: Figure S2.** Heatmaps for 398 risk-related genes and 64 LASSO screened genes. Unsupervised heatmaps for 398 risk-related genes (A) and 64 LASSO screened genes (B); Unsupervised heatmaps where smoking related residual values were removed in the gene expression matrix for 398 risk-related genes (C) and 64 LASSO screened genes (D). OR, Odds Ratio; LASSO, least absolute shrinkage and selection operator

**Additional file 10: Figure S3.** Feature selection of genes representing different patterns. Consensus cumulative distribution function (CDF) (A) and Delta area (B) of consistent cluster analysis of genes through unsupervised clustering; consensus clustering of genes into four clusters (C); GO analysis of four clusters (D); prediction effect of 114 genes into the logistic regression model (classifying high-risk, H and low-risk, L) in training set (E) and external validation set (GSE30219) (F); prediction effect of 41 genes with significant coefficients into the logistic regression model (classifying high-risk, H and low-risk, L) in training set (G) and external validation set (GSE30219) (H); Heatmaps of 114 genes in the training set (original: I, smoking related residual values removed: K) and the validation set (J) respectively; GO analysis of 64 genes of IA score (L); Expression distribution of risk genes or oncogenes ((OR > 1) (M) and protective genes or tumor suppressor genes (OR < 1) (N) in early-stage LUAD (AIS, MIA and IAC). CDF, Consensus cumulative distribution function, OR, Odds Ratio; LASSO, least absolute shrinkage and selection operator; LUAD, lung adenocarcinoma; carcinoma in situ (AIS), microinvasive adenocarcinoma (MIA), invasive adenocarcinoma (IAC)

**Additional file 11: Figure S4.** The effect of IA risk classification in the 8th TNM staging and the indication of IA score for chemotherapy sensitivity or not. (A) OS (left) and DSS (right) curves among IA1, IA2, IA3 and IB-II LUAD patients in SEER database; (B) OS (left) and DSS (right) curves between two categories of stage IA LUAD patients divided by receiving chemotherapy or not in SEER database (Some IA patients have no defined subclass); (C) OS (left) and DFS (right) curves between two categories of stage IA LUAD patients divided by receiving chemotherapy or not in GEO datasets possessing data about chemotherapy or not (GSE13213, GSE31210, GSE42127, GSE68465); (D) OS (left) and DSS (right) curves between two categories of stage IA3 LUAD patients divided by receiving chemotherapy or not in SEER database; (E) OS (left) and DFS (right) curves between two categories of stage IA L (low-risk type classified by IA score) LUAD patients divided by receiving chemotherapy or not in GEO datasets; (F) OS (left) and DFS (right) curves between two categories of stage IA H (high-risk type classified by IA score) LUAD patients divided by receiving chemotherapy or not in GEO datasets; (G) OS (left) and DSS (right) curves between two categories of stage IB-II LUAD patients divided by receiving chemotherapy or not in SEER database; (H) OS (left) and DFS (right) curves between two categories of stage IB-II LUAD patients divided by receiving chemotherapy or not in GEO datasets possessing data about chemotherapy or not; (I) OS (left) and DFS (right) curves between two categories of stage IB-II L (low-risk type classified by IA score) LUAD patients divided by receiving chemotherapy or not in GEO datasets; (J) OS (left) and DFS (right) curves between two categories of stage IB-II H (high-risk type classified by IA score) LUAD patients divided by receiving chemotherapy or not in GEO datasets. LUAD, lung adenocarcinoma; OS, overall survival; DFS, disease-free survival; DSS; disease-specific survival; SEER, Surveillance Epidemiology and End Results

**Additional file 12.** Supplementary text.

## Data Availability

Original data can be found in https://www.ncbi.nlm.nih.gov/geo/, https://portal.gdc.cancer.gov/, https://ngdc.cncb.ac.cn/gsa-human, and https://seer.cancer.gov/.

## Abbreviations

LUAD: Lung adenocarcinoma
OS: Overall survival
SEER: Surveillance Epidemiology and End Results
TCGA: The Cancer Genome Atlas
GSEA: Gene Set Enrichment Analysis
CNV: Copy number variation
gbm: Stochastic Gradient Boosting
rf: Random Forest
knn: K-Nearest Neighbor
nb: Naive Bayes
nnet: Neural Network
pls: Partial Least Squares
svm: Support Vector Machine
treebag: Bagged Classification And Regression Tree
xgbTree: Extreme Gradient Boosting
LASSO: Least absolute shrinkage and selection operator
BH: Benjamini–Hochberg
ROC: Receiver operating characteristic
AUC: Area under the curve
DFS: Disease-free survival
DSS: Disease-specific survival
DEGs: Differentially expressed genes
RTK: Receptor tyrosine kinase
MAD: Median absolute deviation
UQ: Upper quartile
NCCN: National Comprehensive Cancer Network
CDF: Cumulative distribution function
AIS: Carcinoma in situ
MIA: Microinvasive adenocarcinoma
IAC: Invasive adenocarcinoma
