# Supplementary material for: A gene-based score for the risk stratification of stage IA lung adenocarcinoma
Source: Respir Res. 2024 Jan 4;25:18. doi: 10.1186/s12931-023-02647-4 (PMC10765678; doi:10.1186/s12931-023-02647-4)
Supplement: Supplementary file 1 — Additional file 1: Table S1. Details of the public datasets in this study. [file 12931_2023_2647_MOESM1_ESM.docx]

| **Details of the public datasets in this study** | | | | | | | | | | |
| --- | --- | --- | --- | --- | --- | --- | --- | --- | --- | --- |
| **Series** | **Platform or Methods** | **Normalization** | **Histological type** | | | **Clinical stage** | | | | **Reference** |
|  |  |  |  |  |  | **(LUAD with detailed records)** | | | |  |
|  |  |  | **AD** | **SQC** | **Normal** | **IA** | **IB-II** | **III** | **IV** |  |
| **GSE13213** | **GPL6480** | **LOWESS normalized** | **117** | **0** | **0** | **42** | **50** | **25** | **0** | **1** |
| **GSE26939** | **GPL9053** | **loess normalization** | **116** | **0** | **0** | **31** | **50** | **19** | **2** | **2** |
| **GSE30219** | **GPL570** | **Robust Multi-Array average (RMA) algorithm** | **85** | **61** | **14** | **71** | **13** | **1** | **0** | **3** |
| **GSE31210** | **GPL570** | **MAS5** | **204** | **0** | **20** | **109** | **95** | **0** | **0** | **4** |
| **GSE41271** | **GPL6884** | **MBCB algorithm** | **183** | **80** | **0** | **36** | **92** | **49** | **4** | **5** |
| **GSE42127** | **GPL6884** | **Model-Based Background Correction (MBCB) method** | **133** | **43** | **0** | **32** | **79** | **20** | **1** | **6** |
| **GSE50081** | **GPL570** | **RMA** | **127** | **43** | **0** | **36** | **91** | **0** | **0** | **7** |
| **GSE63459** | **GPL6883** | **RSN-normalized** | **33** | **0** | **32** | **17** | **15** | **0** | **0** | **8** |
| **GSE68465** | **GPL96** | **MAS5** | **443** | **0** | **19** | **115** | **257** | **68** | **0** | **9** |
| **GSE68571** | **GPL80** | **Z-scaling** | **86** | **0** | **10** | **67** | **4** | **15** | **0** | **10** |
| **GSE72094** | **GPL15048** | **IRON normalization** | **442** | **0** | **0** | **150** | **166** | **57** | **15** | **11** |
| **TCGA-LUAD** | **Illumina HiSeq** | **upper quartile normalized RSEM count** | **522** | **0** | **59** | **131** | **255** | **81** | **26** | **12** |
| **Reference:** | | | | | | | | | | |
| **1. Tomida S, Takeuchi T, Shimada Y, et al. Relapse-related molecular signature in lung adenocarcinomas identifies patients with dismal prognosis. Journal of clinical oncology : official journal of the American Society of Clinical Oncology. 2009;27(17):2793-2799.** | | | | | | | | | | |
| **2. Wilkerson MD, Yin X, Walter V, et al. Differential pathogenesis of lung adenocarcinoma subtypes involving sequence mutations, copy number, chromosomal instability, and methylation. PloS one. 2012;7(5):e36530.** | | | | | | | | | | |
| **3. Rousseaux S, Debernardi A, Jacquiau B, et al. Ectopic activation of germline and placental genes identifies aggressive metastasis-prone lung cancers. Sci Transl Med. 2013;5(186):186ra166.** | | | | | | | | | | |
| **4. Okayama H, Kohno T, Ishii Y, et al. Identification of genes upregulated in ALK-positive and EGFR/KRAS/ALK-negative lung adenocarcinomas. Cancer Res. 2012;72(1):100-111.** | | | | | | | | | | |
| **5. Sato M, Larsen JE, Lee W, et al. Human lung epithelial cells progressed to malignancy through specific oncogenic manipulations. Mol. Cancer Res. 2013;11(6):638-650.** | | | | | | | | | | |
| **6. Tang H, Xiao G, Behrens C, et al. A 12-gene set predicts survival benefits from adjuvant chemotherapy in non-small cell lung cancer patients. Clinical cancer research : an official journal of the American Association for Cancer Research. 2013;19(6):1577-1586.** | | | | | | | | | | |
| **7. Der SD, Sykes J, Pintilie M, et al. Validation of a histology-independent prognostic gene signature for early-stage, non-small-cell lung cancer including stage IA patients. J Thorac Oncol. 2014;9(1):59-64.** | | | | | | | | | | |
| **8. Robles AI, Arai E, Mathé EA, et al. An Integrated Prognostic Classifier for Stage I Lung Adenocarcinoma Based on mRNA, microRNA, and DNA Methylation Biomarkers. J Thorac Oncol. 2015;10(7):1037-1048.** | | | | | | | | | | |
| **9. Shedden K, Taylor JM, Enkemann SA, et al. Gene expression-based survival prediction in lung adenocarcinoma: a multi-site, blinded validation study. Nature medicine. 2008;14(8):822-827.** | | | | | | | | | | |
| **10. Beer DG, Kardia SL, Huang CC, et al. Gene-expression profiles predict survival of patients with lung adenocarcinoma. Nature medicine. 2002;8(8):816-824.** | | | | | | | | | | |
| **11. Schabath MB, Welsh EA, Fulp WJ, et al. Differential association of STK11 and TP53 with KRAS mutation-associated gene expression, proliferation and immune surveillance in lung adenocarcinoma. Oncogene. 2016;35(24):3209-3216.** | | | | | | | | | | |
| **12. Weinstein JN, Collisson EA, Mills GB, et al. The Cancer Genome Atlas Pan-Cancer analysis project. Nature genetics. 2013;45(10):1113-1120.** | | | | | | | | | | |
