# Supplementary material for: A gene-based score for the risk stratification of stage IA lung adenocarcinoma
Source: Respir Res. 2024 Jan 4;25:18. doi: 10.1186/s12931-023-02647-4 (PMC10765678; doi:10.1186/s12931-023-02647-4)
Supplement: Supplementary file 2 — Additional file 2: Table S2. General information for fivefold cross-validation of stage IA in training set. [file 12931_2023_2647_MOESM2_ESM.docx]

| **Table S2. General information for 5-fold cross-validation of stage IA in training set** | | | | | | | | | |  |
| --- | --- | --- | --- | --- | --- | --- | --- | --- | --- | --- |
|  |  |  |  |  |  |  |  |  |  |  |
|  | **K1** | | **K2** | | **K3** | | **K4** | | **K5** |  |
| **Survival status (high-risk vs. low-risk)** | | **0.618 (118 vs. 191)** | | **0.537 (108 vs. 201)** | | **0.553 (110 vs. 199)** | | **0.5 (103 vs. 206)** | **0.579 (113 vs. 195)** |  |
| **Gender ratio (female vs. male)** | | **1.255 (172 vs. 137)** | | **1.433 (182 vs. 127)** | | **1.377 (179 vs. 130)** | | **1.341 (177 vs. 132)** | **1.369 (178 vs. 130)** |  |
| **Age median (min, max)** | | **63.50 (34, 88)** | | **63 (35, 88)** | | **63 (34, 87)** | | **64 (34, 88)** | **64 (34, 88)** |  |
| **Smoking status (yes vs. no)** | | **3.266 (209 vs. 64)** | | **3.045 (201 vs. 66)** | | **3.377 (206 vs. 61)** | | **3.108 (202 vs. 65)** | **3.094 (198 vs. 64)** |  |
