# Supplementary material for: A gene-based score for the risk stratification of stage IA lung adenocarcinoma
Source: Respir Res. 2024 Jan 4;25:18. doi: 10.1186/s12931-023-02647-4 (PMC10765678; doi:10.1186/s12931-023-02647-4)
Supplement: Supplementary file 3 — Additional file 3: Table S3. Gene symbols and gene ids for 398 risk-related genes. [file 12931_2023_2647_MOESM3_ESM.docx]

| **Number** | **ENTREZID** | **SYMBOL** |
| --- | --- | --- |
| **1** | **1000** | **CDH2** |
| **2** | **10024** | **TROAP** |
| **3** | **10093** | **ARPC4** |
| **4** | **10105** | **PPIF** |
| **5** | **1017** | **CDK2** |
| **6** | **10184** | **LHFPL2** |
| **7** | **10213** | **PSMD14** |
| **8** | **1033** | **CDKN3** |
| **9** | **10381** | **TUBB3** |
| **10** | **10397** | **NDRG1** |
| **11** | **10426** | **TUBGCP3** |
| **12** | **10488** | **CREB3** |
| **13** | **1054** | **CEBPG** |
| **14** | **10574** | **CCT7** |
| **15** | **1062** | **CENPE** |
| **16** | **1063** | **CENPF** |
| **17** | **1072** | **CFL1** |
| **18** | **10730** | **YME1L1** |
| **19** | **10733** | **PLK4** |
| **20** | **10797** | **MTHFD2** |
| **21** | **10859** | **LILRB1** |
| **22** | **10874** | **NMU** |
| **23** | **10897** | **YIF1A** |
| **24** | **10962** | **MLLT11** |
| **25** | **10993** | **SDS** |
| **26** | **11004** | **KIF2C** |
| **27** | **11047** | **ADRM1** |
| **28** | **11065** | **UBE2C** |
| **29** | **11082** | **ESM1** |
| **30** | **1158** | **CKM** |
| **31** | **1163** | **CKS1B** |
| **32** | **1173** | **AP2M1** |
| **33** | **1175** | **AP2S1** |
| **34** | **1192** | **CLIC1** |
| **35** | **133** | **ADM** |
| **36** | **1340** | **COX6B1** |
| **37** | **1347** | **COX7A2** |
| **38** | **1435** | **CSF1** |
| **39** | **1476** | **CSTB** |
| **40** | **1478** | **CSTF2** |
| **41** | **1508** | **CTSB** |
| **42** | **1603** | **DAD1** |
| **43** | **1824** | **DSC2** |
| **44** | **1841** | **DTYMK** |
| **45** | **1869** | **E2F1** |
| **46** | **1871** | **E2F3** |
| **47** | **1944** | **EFNA3** |
| **48** | **1965** | **EIF2S1** |
| **49** | **215** | **ABCD1** |
| **50** | **2175** | **FANCA** |
| **51** | **222** | **ALDH3B2** |
| **52** | **226** | **ALDOA** |
| **53** | **22919** | **MAPRE1** |
| **54** | **2305** | **FOXM1** |
| **55** | **23204** | **ARL6IP1** |
| **56** | **23310** | **NCAPD3** |
| **57** | **2633** | **GBP1** |
| **58** | **2650** | **GCNT1** |
| **59** | **2717** | **GLA** |
| **60** | **27257** | **LSM1** |
| **61** | **2730** | **GCLM** |
| **62** | **27338** | **UBE2S** |
| **63** | **2773** | **GNAI3** |
| **64** | **2784** | **GNB3** |
| **65** | **2821** | **GPI** |
| **66** | **2842** | **GPR19** |
| **67** | **2919** | **CXCL1** |
| **68** | **29893** | **PSMC3IP** |
| **69** | **2999** | **GZMH** |
| **70** | **3066** | **HDAC2** |
| **71** | **30836** | **DNTTIP2** |
| **72** | **3099** | **HK2** |
| **73** | **3148** | **HMGB2** |
| **74** | **3161** | **HMMR** |
| **75** | **3162** | **HMOX1** |
| **76** | **3181** | **HNRNPA2B1** |
| **77** | **332** | **BIRC5** |
| **78** | **3421** | **IDH3G** |
| **79** | **3428** | **IFI16** |
| **80** | **3484** | **IGFBP1** |
| **81** | **3486** | **IGFBP3** |
| **82** | **3553** | **IL1B** |
| **83** | **3608** | **ILF2** |
| **84** | **3627** | **CXCL10** |
| **85** | **3659** | **IRF1** |
| **86** | **3678** | **ITGA5** |
| **87** | **3832** | **KIF11** |
| **88** | **3833** | **KIFC1** |
| **89** | **3837** | **KPNB1** |
| **90** | **3838** | **KPNA2** |
| **91** | **3853** | **KRT6A** |
| **92** | **3856** | **KRT8** |
| **93** | **3868** | **KRT16** |
| **94** | **3875** | **KRT18** |
| **95** | **3880** | **KRT19** |
| **96** | **389** | **RHOC** |
| **97** | **390** | **RND3** |
| **98** | **3925** | **STMN1** |
| **99** | **3939** | **LDHA** |
| **100** | **3948** | **LDHC** |
| **101** | **3956** | **LGALS1** |
| **102** | **4001** | **LMNB1** |
| **103** | **4017** | **LOXL2** |
| **104** | **4067** | **LYN** |
| **105** | **4082** | **MARCKS** |
| **106** | **4084** | **MXD1** |
| **107** | **4085** | **MAD2L1** |
| **108** | **4151** | **MB** |
| **109** | **4171** | **MCM2** |
| **110** | **4172** | **MCM3** |
| **111** | **4173** | **MCM4** |
| **112** | **4174** | **MCM5** |
| **113** | **4175** | **MCM6** |
| **114** | **4282** | **MIF** |
| **115** | **4288** | **MKI67** |
| **116** | **4318** | **MMP9** |
| **117** | **4521** | **NUDT1** |
| **118** | **4605** | **MYBL2** |
| **119** | **4647** | **MYO7A** |
| **120** | **4691** | **NCL** |
| **121** | **4704** | **NDUFA9** |
| **122** | **4738** | **NEDD8** |
| **123** | **4751** | **NEK2** |
| **124** | **483** | **ATP1B3** |
| **125** | **4830** | **NME1** |
| **126** | **4893** | **NRAS** |
| **127** | **5031** | **P2RY6** |
| **128** | **5033** | **P4HA1** |
| **129** | **5111** | **PCNA** |
| **130** | **5130** | **PCYT1A** |
| **131** | **5210** | **PFKFB4** |
| **132** | **5214** | **PFKP** |
| **133** | **5217** | **PFN2** |
| **134** | **5223** | **PGAM1** |
| **135** | **5268** | **SERPINB5** |
| **136** | **5307** | **PITX1** |
| **137** | **5328** | **PLAU** |
| **138** | **5329** | **PLAUR** |
| **139** | **5347** | **PLK1** |
| **140** | **5352** | **PLOD2** |
| **141** | **5366** | **PMAIP1** |
| **142** | **5437** | **POLR2H** |
| **143** | **5557** | **PRIM1** |
| **144** | **5653** | **KLK6** |
| **145** | **5686** | **PSMA5** |
| **146** | **56902** | **PNO1** |
| **147** | **5691** | **PSMB3** |
| **148** | **5693** | **PSMB5** |
| **149** | **5695** | **PSMB7** |
| **150** | **5698** | **PSMB9** |
| **151** | **5707** | **PSMD1** |
| **152** | **5715** | **PSMD9** |
| **153** | **5720** | **PSME1** |
| **154** | **5724** | **PTAFR** |
| **155** | **5836** | **PYGL** |
| **156** | **5982** | **RFC2** |
| **157** | **5984** | **RFC4** |
| **158** | **5985** | **RFC5** |
| **159** | **6241** | **RRM2** |
| **160** | **6278** | **S100A7** |
| **161** | **6279** | **S100A8** |
| **162** | **6280** | **S100A9** |
| **163** | **6281** | **S100A10** |
| **164** | **6282** | **S100A11** |
| **165** | **6283** | **S100A12** |
| **166** | **6286** | **S100P** |
| **167** | **6364** | **CCL20** |
| **168** | **6373** | **CXCL11** |
| **169** | **641** | **BLM** |
| **170** | **6452** | **SH3BP2** |
| **171** | **6484** | **ST3GAL4** |
| **172** | **6491** | **STIL** |
| **173** | **6513** | **SLC2A1** |
| **174** | **6520** | **SLC3A2** |
| **175** | **6535** | **SLC6A8** |
| **176** | **6566** | **SLC16A1** |
| **177** | **6574** | **SLC20A1** |
| **178** | **6617** | **SNAPC1** |
| **179** | **6627** | **SNRPA1** |
| **180** | **6632** | **SNRPD1** |
| **181** | **6633** | **SNRPD2** |
| **182** | **6636** | **SNRPF** |
| **183** | **6696** | **SPP1** |
| **184** | **6699** | **SPRR1B** |
| **185** | **672** | **BRCA1** |
| **186** | **6772** | **STAT1** |
| **187** | **6890** | **TAP1** |
| **188** | **6999** | **TDO2** |
| **189** | **7032** | **TFF2** |
| **190** | **7039** | **TGFA** |
| **191** | **7083** | **TK1** |
| **192** | **7112** | **TMPO** |
| **193** | **7130** | **TNFAIP6** |
| **194** | **7153** | **TOP2A** |
| **195** | **7170** | **TPM3** |
| **196** | **7247** | **TSN** |
| **197** | **7272** | **TTK** |
| **198** | **7277** | **TUBA4A** |
| **199** | **7283** | **TUBG1** |
| **200** | **7298** | **TYMS** |
| **201** | **7316** | **UBC** |
| **202** | **7319** | **UBE2A** |
| **203** | **7336** | **UBE2V2** |
| **204** | **7347** | **UCHL3** |
| **205** | **7368** | **UGT8** |
| **206** | **7408** | **VASP** |
| **207** | **7416** | **VDAC1** |
| **208** | **7422** | **VEGFA** |
| **209** | **7444** | **VRK2** |
| **210** | **7490** | **WT1** |
| **211** | **7520** | **XRCC5** |
| **212** | **7533** | **YWHAH** |
| **213** | **768** | **CA9** |
| **214** | **7837** | **PXDN** |
| **215** | **7850** | **IL1R2** |
| **216** | **7884** | **SLBP** |
| **217** | **79173** | **C19orf57** |
| **218** | **8061** | **FOSL1** |
| **219** | **8078** | **USP5** |
| **220** | **8140** | **SLC7A5** |
| **221** | **81569** | **ACTL8** |
| **222** | **8270** | **LAGE3** |
| **223** | **8317** | **CDC7** |
| **224** | **84804** | **MFSD9** |
| **225** | **84823** | **LMNB2** |
| **226** | **8638** | **OASL** |
| **227** | **8655** | **DYNLL1** |
| **228** | **8754** | **ADAM9** |
| **229** | **8836** | **GGH** |
| **230** | **890** | **CCNA2** |
| **231** | **891** | **CCNB1** |
| **232** | **8942** | **KYNU** |
| **233** | **899** | **CCNF** |
| **234** | **9088** | **PKMYT1** |
| **235** | **9168** | **TMSB10** |
| **236** | **9319** | **TRIP13** |
| **237** | **9355** | **LHX2** |
| **238** | **9493** | **KIF23** |
| **239** | **9650** | **MTFR1** |
| **240** | **9688** | **NUP93** |
| **241** | **9775** | **EIF4A3** |
| **242** | **978** | **CDA** |
| **243** | **9833** | **MELK** |
| **244** | **990** | **CDC6** |
| **245** | **991** | **CDC20** |
| **246** | **9928** | **KIF14** |
| **247** | **993** | **CDC25A** |
| **248** | **995** | **CDC25C** |
| **249** | **9982** | **FGFBP1** |
| **250** | **10129** | **FRY** |
| **251** | **10181** | **RBM5** |
| **252** | **10370** | **CITED2** |
| **253** | **104** | **ADARB1** |
| **254** | **10577** | **NPC2** |
| **255** | **10810** | **WASF3** |
| **256** | **10902** | **BRD8** |
| **257** | **11343** | **MGLL** |
| **258** | **1153** | **CIRBP** |
| **259** | **1285** | **COL4A3** |
| **260** | **1286** | **COL4A4** |
| **261** | **1359** | **CPA3** |
| **262** | **1471** | **CST3** |
| **263** | **1511** | **CTSG** |
| **264** | **1512** | **CTSH** |
| **265** | **1580** | **CYP4B1** |
| **266** | **1604** | **CD55** |
| **267** | **1793** | **DOCK1** |
| **268** | **1808** | **DPYSL2** |
| **269** | **1938** | **EEF2** |
| **270** | **1949** | **EFNB3** |
| **271** | **2006** | **ELN** |
| **272** | **203** | **AK1** |
| **273** | **2033** | **EP300** |
| **274** | **2057** | **EPOR** |
| **275** | **2060** | **EPS15** |
| **276** | **2066** | **ERBB4** |
| **277** | **2119** | **ETV5** |
| **278** | **2145** | **EZH1** |
| **279** | **2159** | **F10** |
| **280** | **2166** | **FAAH** |
| **281** | **2217** | **FCGRT** |
| **282** | **224** | **ALDH3A2** |
| **283** | **2273** | **FHL1** |
| **284** | **2308** | **FOXO1** |
| **285** | **23171** | **GPD1L** |
| **286** | **2327** | **FMO2** |
| **287** | **23479** | **ISCU** |
| **288** | **2348** | **FOLR1** |
| **289** | **2517** | **FUCA1** |
| **290** | **25802** | **LMOD1** |
| **291** | **2662** | **GDF10** |
| **292** | **2690** | **GHR** |
| **293** | **26999** | **CYFIP2** |
| **294** | **2719** | **GPC3** |
| **295** | **2735** | **GLI1** |
| **296** | **275** | **AMT** |
| **297** | **2824** | **GPM6B** |
| **298** | **284** | **ANGPT1** |
| **299** | **2908** | **NR3C1** |
| **300** | **2949** | **GSTM5** |
| **301** | **3067** | **HDC** |
| **302** | **3104** | **ZBTB48** |
| **303** | **3131** | **HLF** |
| **304** | **3249** | **HPN** |
| **305** | **3483** | **IGFALS** |
| **306** | **350** | **APOH** |
| **307** | **3570** | **IL6R** |
| **308** | **3572** | **IL6ST** |
| **309** | **3598** | **IL13RA2** |
| **310** | **36** | **ACADSB** |
| **311** | **360** | **AQP3** |
| **312** | **3700** | **ITIH4** |
| **313** | **3708** | **ITPR1** |
| **314** | **3709** | **ITPR2** |
| **315** | **373** | **TRIM23** |
| **316** | **3772** | **KCNJ15** |
| **317** | **38** | **ACAT1** |
| **318** | **3908** | **LAMA2** |
| **319** | **3931** | **LCAT** |
| **320** | **4091** | **SMAD6** |
| **321** | **4122** | **MAN2A2** |
| **322** | **4128** | **MAOA** |
| **323** | **4215** | **MAP3K3** |
| **324** | **4239** | **MFAP4** |
| **325** | **4256** | **MGP** |
| **326** | **445** | **ASS1** |
| **327** | **4628** | **MYH10** |
| **328** | **4629** | **MYH11** |
| **329** | **4641** | **MYO1C** |
| **330** | **4673** | **NAP1L1** |
| **331** | **4784** | **NFIX** |
| **332** | **4882** | **NPR2** |
| **333** | **4908** | **NTF3** |
| **334** | **4948** | **OCA2** |
| **335** | **5225** | **PGC** |
| **336** | **5319** | **PLA2G1B** |
| **337** | **53358** | **SHC3** |
| **338** | **5372** | **PMM1** |
| **339** | **5446** | **PON3** |
| **340** | **5564** | **PRKAB1** |
| **341** | **55885** | **LMO3** |
| **342** | **57326** | **PBXIP1** |
| **343** | **5813** | **PURA** |
| **344** | **590** | **BCHE** |
| **345** | **5909** | **RAP1GAP** |
| **346** | **6035** | **RNASE1** |
| **347** | **6095** | **RORA** |
| **348** | **6103** | **RPGR** |
| **349** | **6135** | **RPL11** |
| **350** | **6196** | **RPS6KA2** |
| **351** | **6439** | **SFTPB** |
| **352** | **6448** | **SGSH** |
| **353** | **6595** | **SMARCA2** |
| **354** | **6604** | **SMARCD3** |
| **355** | **6651** | **SON** |
| **356** | **6786** | **STIM1** |
| **357** | **6792** | **CDKL5** |
| **358** | **6812** | **STXBP1** |
| **359** | **6844** | **VAMP2** |
| **360** | **6909** | **TBX2** |
| **361** | **6919** | **TCEA2** |
| **362** | **7048** | **TGFBR2** |
| **363** | **7049** | **TGFBR3** |
| **364** | **7080** | **NKX2-1** |
| **365** | **7089** | **TLE2** |
| **366** | **7102** | **TSPAN7** |
| **367** | **7189** | **TRAF6** |
| **368** | **722** | **C4BPA** |
| **369** | **730** | **C7** |
| **370** | **7306** | **TYRP1** |
| **371** | **7356** | **SCGB1A1** |
| **372** | **7375** | **USP4** |
| **373** | **7431** | **VIM** |
| **374** | **7433** | **VIPR1** |
| **375** | **7508** | **XPC** |
| **376** | **7551** | **ZNF3** |
| **377** | **7799** | **PRDM2** |
| **378** | **7867** | **MAPKAPK3** |
| **379** | **7915** | **ALDH5A1** |
| **380** | **81576** | **CCDC130** |
| **381** | **8309** | **ACOX2** |
| **382** | **847** | **CAT** |
| **383** | **8516** | **ITGA8** |
| **384** | **8554** | **PIAS1** |
| **385** | **8558** | **CDK10** |
| **386** | **857** | **CAV1** |
| **387** | **8578** | **SCARF1** |
| **388** | **8659** | **ALDH4A1** |
| **389** | **8729** | **GBF1** |
| **390** | **8814** | **CDKL1** |
| **391** | **8848** | **TSC22D1** |
| **392** | **8925** | **HERC1** |
| **393** | **896** | **CCND3** |
| **394** | **8991** | **SELENBP1** |
| **395** | **9459** | **ARHGEF6** |
| **396** | **9514** | **GAL3ST1** |
| **397** | **9663** | **LPIN2** |
| **398** | **975** | **CD81** |
