# Supplementary material for: A gene-based score for the risk stratification of stage IA lung adenocarcinoma
Source: Respir Res. 2024 Jan 4;25:18. doi: 10.1186/s12931-023-02647-4 (PMC10765678; doi:10.1186/s12931-023-02647-4)
Supplement: Supplementary file 4 — Additional file 4: Table S4. Gene symbols and gene ids for 64 LASSO screened genes. [file 12931_2023_2647_MOESM4_ESM.docx]

| **Number** | **ENTREZID** | **SYMBOL** |
| --- | --- | --- |
| **1** | **1000** | **CDH2** |
| **2** | **10397** | **NDRG1** |
| **3** | **10426** | **TUBGCP3** |
| **4** | **10488** | **CREB3** |
| **5** | **1062** | **CENPE** |
| **6** | **1072** | **CFL1** |
| **7** | **10874** | **NMU** |
| **8** | **1158** | **CKM** |
| **9** | **1435** | **CSF1** |
| **10** | **1949** | **EFNB3** |
| **11** | **1965** | **EIF2S1** |
| **12** | **2060** | **EPS15** |
| **13** | **2119** | **ETV5** |
| **14** | **215** | **ABCD1** |
| **15** | **226** | **ALDOA** |
| **16** | **2308** | **FOXO1** |
| **17** | **23204** | **ARL6IP1** |
| **18** | **2517** | **FUCA1** |
| **19** | **27257** | **LSM1** |
| **20** | **2784** | **GNB3** |
| **21** | **2919** | **CXCL1** |
| **22** | **2999** | **GZMH** |
| **23** | **3131** | **HLF** |
| **24** | **3181** | **HNRNPA2B1** |
| **25** | **3421** | **IDH3G** |
| **26** | **3484** | **IGFBP1** |
| **27** | **3598** | **IL13RA2** |
| **28** | **3700** | **ITIH4** |
| **29** | **38** | **ACAT1** |
| **30** | **3875** | **KRT18** |
| **31** | **3948** | **LDHC** |
| **32** | **4017** | **LOXL2** |
| **33** | **4067** | **LYN** |
| **34** | **4128** | **MAOA** |
| **35** | **445** | **ASS1** |
| **36** | **4647** | **MYO7A** |
| **37** | **4673** | **NAP1L1** |
| **38** | **5653** | **KLK6** |
| **39** | **6035** | **RNASE1** |
| **40** | **6283** | **S100A12** |
| **41** | **6448** | **SGSH** |
| **42** | **6535** | **SLC6A8** |
| **43** | **6595** | **SMARCA2** |
| **44** | **7032** | **TFF2** |
| **45** | **7170** | **TPM3** |
| **46** | **7189** | **TRAF6** |
| **47** | **7316** | **UBC** |
| **48** | **7368** | **UGT8** |
| **49** | **7551** | **ZNF3** |
| **50** | **7799** | **PRDM2** |
| **51** | **7837** | **PXDN** |
| **52** | **7850** | **IL1R2** |
| **53** | **7867** | **MAPKAPK3** |
| **54** | **79173** | **C19orf57** |
| **55** | **81569** | **ACTL8** |
| **56** | **81576** | **CCDC130** |
| **57** | **84804** | **MFSD9** |
| **58** | **8638** | **OASL** |
| **59** | **8729** | **GBF1** |
| **60** | **8754** | **ADAM9** |
| **61** | **9319** | **TRIP13** |
| **62** | **9514** | **GAL3ST1** |
| **63** | **9663** | **LPIN2** |
| **64** | **9928** | **KIF14** |
