# Supplementary material for: A gene-based score for the risk stratification of stage IA lung adenocarcinoma
Source: Respir Res. 2024 Jan 4;25:18. doi: 10.1186/s12931-023-02647-4 (PMC10765678; doi:10.1186/s12931-023-02647-4)
Supplement: Supplementary file 5 — Additional file 5: Table S5. Gene symbols and gene ids for 114 genes by LASSO within gene clusters. [file 12931_2023_2647_MOESM5_ESM.docx]

| **Number** | **ENTREZID** | **SYMBOL** |
| --- | --- | --- |
| **1** | **1000** | **CDH2** |
| **2** | **10397** | **NDRG1** |
| **3** | **10426** | **TUBGCP3** |
| **4** | **10488** | **CREB3** |
| **5** | **10874** | **NMU** |
| **6** | **1158** | **CKM** |
| **7** | **1435** | **CSF1** |
| **8** | **1949** | **EFNB3** |
| **9** | **2033** | **EP300** |
| **10** | **215** | **ABCD1** |
| **11** | **2735** | **GLI1** |
| **12** | **2784** | **GNB3** |
| **13** | **2919** | **CXCL1** |
| **14** | **2999** | **GZMH** |
| **15** | **3421** | **IDH3G** |
| **16** | **3484** | **IGFBP1** |
| **17** | **3700** | **ITIH4** |
| **18** | **3875** | **KRT18** |
| **19** | **3948** | **LDHC** |
| **20** | **4067** | **LYN** |
| **21** | **4641** | **MYO1C** |
| **22** | **4647** | **MYO7A** |
| **23** | **4673** | **NAP1L1** |
| **24** | **4908** | **NTF3** |
| **25** | **5653** | **KLK6** |
| **26** | **6283** | **S100A12** |
| **27** | **6452** | **SH3BP2** |
| **28** | **6535** | **SLC6A8** |
| **29** | **6699** | **SPRR1B** |
| **30** | **7032** | **TFF2** |
| **31** | **7170** | **TPM3** |
| **32** | **7189** | **TRAF6** |
| **33** | **7316** | **UBC** |
| **34** | **7368** | **UGT8** |
| **35** | **7375** | **USP4** |
| **36** | **7422** | **VEGFA** |
| **37** | **7520** | **XRCC5** |
| **38** | **7551** | **ZNF3** |
| **39** | **7837** | **PXDN** |
| **40** | **79173** | **C19orf57** |
| **41** | **81569** | **ACTL8** |
| **42** | **8638** | **OASL** |
| **43** | **8659** | **ALDH4A1** |
| **44** | **8729** | **GBF1** |
| **45** | **8754** | **ADAM9** |
| **46** | **9663** | **LPIN2** |
| **47** | **1871** | **E2F3** |
| **48** | **1965** | **EIF2S1** |
| **49** | **226** | **ALDOA** |
| **50** | **23204** | **ARL6IP1** |
| **51** | **2633** | **GBP1** |
| **52** | **27257** | **LSM1** |
| **53** | **2842** | **GPR19** |
| **54** | **29893** | **PSMC3IP** |
| **55** | **3181** | **HNRNPA2B1** |
| **56** | **3939** | **LDHA** |
| **57** | **5366** | **PMAIP1** |
| **58** | **5836** | **PYGL** |
| **59** | **6696** | **SPP1** |
| **60** | **8270** | **LAGE3** |
| **61** | **9168** | **TMSB10** |
| **62** | **9775** | **EIF4A3** |
| **63** | **10370** | **CITED2** |
| **64** | **1511** | **CTSG** |
| **65** | **1938** | **EEF2** |
| **66** | **2060** | **EPS15** |
| **67** | **2119** | **ETV5** |
| **68** | **2145** | **EZH1** |
| **69** | **2308** | **FOXO1** |
| **70** | **25802** | **LMOD1** |
| **71** | **2662** | **GDF10** |
| **72** | **275** | **AMT** |
| **73** | **2824** | **GPM6B** |
| **74** | **3598** | **IL13RA2** |
| **75** | **3931** | **LCAT** |
| **76** | **4122** | **MAN2A2** |
| **77** | **590** | **BCHE** |
| **78** | **6095** | **RORA** |
| **79** | **6595** | **SMARCA2** |
| **80** | **6604** | **SMARCD3** |
| **81** | **6651** | **SON** |
| **82** | **6909** | **TBX2** |
| **83** | **7049** | **TGFBR3** |
| **84** | **7089** | **TLE2** |
| **85** | **7102** | **TSPAN7** |
| **86** | **730** | **C7** |
| **87** | **7799** | **PRDM2** |
| **88** | **7867** | **MAPKAPK3** |
| **89** | **81576** | **CCDC130** |
| **90** | **8516** | **ITGA8** |
| **91** | **8554** | **PIAS1** |
| **92** | **8814** | **CDKL1** |
| **93** | **8925** | **HERC1** |
| **94** | **10902** | **BRD8** |
| **95** | **1153** | **CIRBP** |
| **96** | **1285** | **COL4A3** |
| **97** | **2066** | **ERBB4** |
| **98** | **2348** | **FOLR1** |
| **99** | **2517** | **FUCA1** |
| **100** | **3104** | **ZBTB48** |
| **101** | **3572** | **IL6ST** |
| **102** | **3709** | **ITPR2** |
| **103** | **373** | **TRIM23** |
| **104** | **3772** | **KCNJ15** |
| **105** | **38** | **ACAT1** |
| **106** | **4128** | **MAOA** |
| **107** | **445** | **ASS1** |
| **108** | **6135** | **RPL11** |
| **109** | **6448** | **SGSH** |
| **110** | **6792** | **CDKL5** |
| **111** | **6919** | **TCEA2** |
| **112** | **8848** | **TSC22D1** |
| **113** | **9514** | **GAL3ST1** |
| **114** | **975** | **CD81** |
