# Supplementary material for: A gene-based score for the risk stratification of stage IA lung adenocarcinoma
Source: Respir Res. 2024 Jan 4;25:18. doi: 10.1186/s12931-023-02647-4 (PMC10765678; doi:10.1186/s12931-023-02647-4)
Supplement: Supplementary file 6 — Additional file 6: Table S6. Risk factors for overall survival in stage IA lung adenocarcinoma. [file 12931_2023_2647_MOESM6_ESM.docx]

| **e-Table 6 Cox proportional hazard regression for overall survival in IA LUAD patients** | | | | | |
| --- | --- | --- | --- | --- | --- |
| **Variable** | **N**  **(848)** | **Median (months)** | **log-rank p** | **Univariate analysis** | **Multivariate analysis** |
|  |  |  |  | **HR (95%) p** | **HR (95%) p** |
|  |  |  |  |  |  |
| **Gender** |  |  | **0.002** | **1.56 (1.17-2.07) 0.002** | **1.99 (1.03-3.85) 0.04** |
| **Male** | **371** | **175** |  |  |  |
| **Female** | **469** | **127** |  |  |  |
| **NA** | **8** |  |  |  |  |
|  |  |  |  |  |  |
| **Age(years)** |  |  | **<0.001** | **1.84 (1.38-2.46) <0.001** | **1.41 (0.76-2.60) 0.28** |
| **>65** | **396** | **175** |  |  |  |
| **<=65** | **443** | **106** |  |  |  |
| **NA** | **9** |  |  |  |  |
|  |  |  |  |  |  |
| **Smoking** |  |  | **<0.001** | **2.38 (1.43-3.96) <0.001** | **1.94 (0.58-6.43) 0.28** |
| **Yes** | **530** | **Not reached** |  |  |  |
| **No** | **144** | **Not reached** |  |  |  |
| **NA** | **174** |  |  |  |  |
|  |  |  |  |  |  |
| **TP53** |  |  | **0.01** | **1.94 (1.17-3.2) 0.01** | **2.36 (1.27-4.37) 0.006** |
| **Mut** | **65** | **71.5** |  |  |  |
| **WT** | **238** | **Not reached** |  |  |  |
| **NA** | **545** |  |  |  |  |
|  |  |  |  |  |  |
| **STK11** |  |  | **0.5** | **0.73 (0.284-1.88) 0.52** |  |
| **Mut** | **24** | **61.7** |  |  |  |
| **WT** | **152** | **72.3** |  |  |  |
| **NA** | **672** |  |  |  |  |
|  |  |  |  |  |  |
| **KRAS** |  |  | **<0.001** | **2.32 (1.44-3.74) <0.001** | **1.40 (0.71-2.79) 0.33** |
| **Mut** | **99** | **Not reached** |  |  |  |
| **WT** | **334** | **Not reached** |  |  |  |
| **NA** | **415** |  |  |  |  |
|  |  |  |  |  |  |
| **EGFR** |  |  | **<0.001** | **0.32 (0.18-0.57) <0.001** | **0.63 (0.21-1.91) 0.41** |
| **Mut** | **129** | **Not reached** |  |  |  |
| **WT** | **270** | **Not reached** |  |  |  |
| **NA** | **449** |  |  |  |  |
|  |  |  |  |  |  |
| **ALK** |  |  | **0.1** | **3.21 (0.74-14) 0.12** |  |
| **Mut** | **9** | **Not reached** |  |  |  |
| **WT** | **165** | **Not reached** |  |  |  |
| **NA** | **674** |  |  |  |  |
| **IA score** |  |  | **<0.001** | **0.18 (0.12-0.25) <0.001** | **0.22 (0.10-0.51) <0.001** |
| **High** | **420** | **175.0** |  |  |  |
| **Low** | **417** | **79.5** |  |  |  |
| **NA** | **11** |  |  |  |  |

**A total of 848 patients with IA LUAD were used for risk factor analysis, of which 837 patients with complete clinical information and gene expression data were used for gene score modeling.**
