# Supplementary figures and images for: A gene-based score for the risk stratification of stage IA lung adenocarcinoma
Source: Respir Res. 2024 Jan 4;25:18. doi: 10.1186/s12931-023-02647-4 (PMC10765678; doi:10.1186/s12931-023-02647-4)

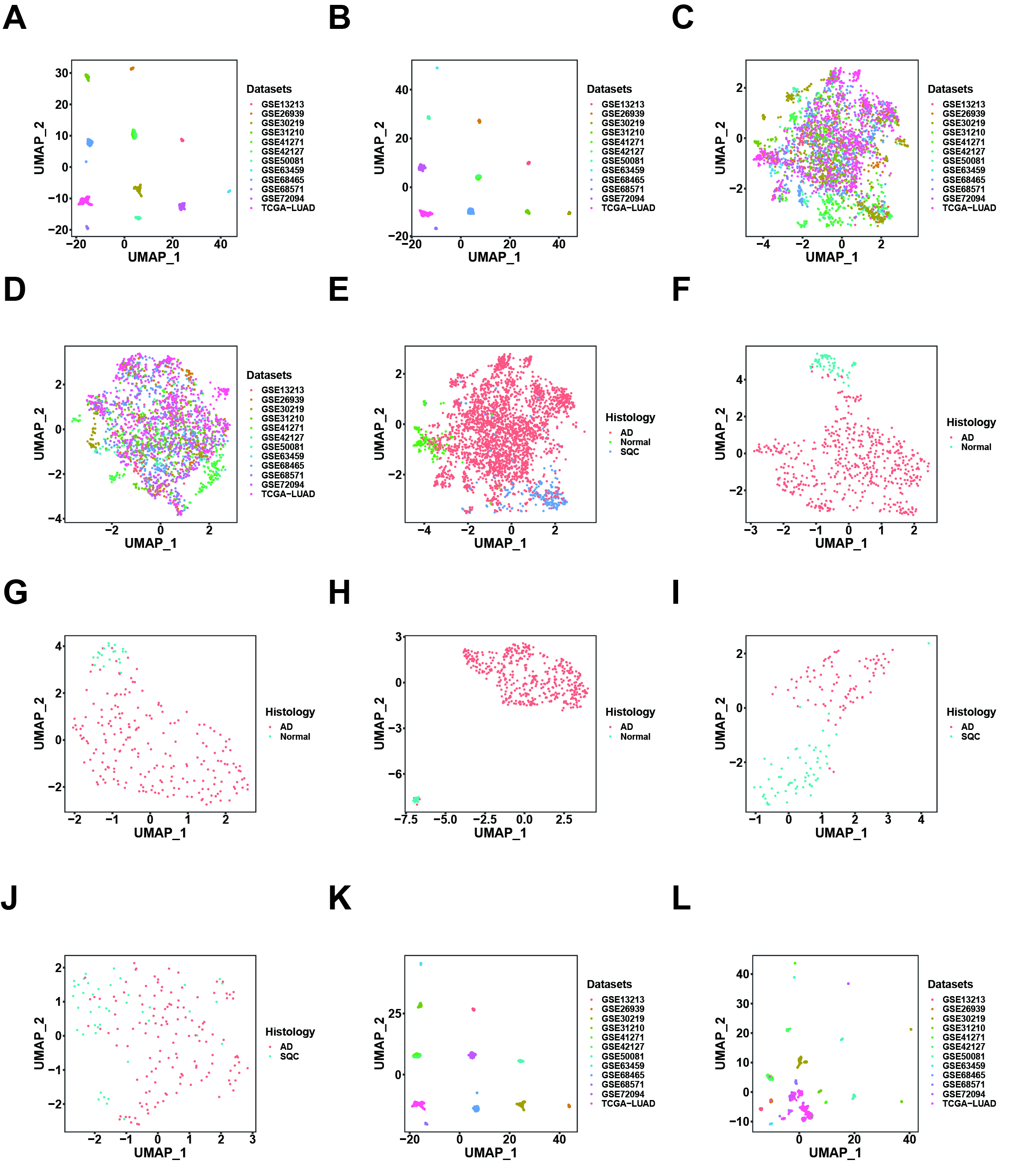

Supplement: Supplementary file 8 — Additional file 8: Figure S1. Analysis of the effect of batch removal of 12 datasets. Usage of U-MAP to exhibit distribution of total samples (A) or LUAD (B) in 12 datasets calculated by gene expression profiles without Z-scaling for normalization; Usage of U-MAP to exhibit distribution of total samples (C) or LUAD (D) in 12 datasets calculated by gene expression profiles with Z-scaling for normalization; Usage of U-MAP to exhibit distribution of different histological sample in total 12 datasets (E) and specific single datasets (F, TCGA-LUAD; G, GSE31210, H, GSE68465; I, GSE30219; J, GSE42127) by gene expression profiles with Z-scaling for normalization. Usage of U-MAP to exhibit distribution of total samples in 12 datasets calculated by gene expression profiles by removeBatchEffect (K) and Combat (L) methods for batch effect removal. LUAD, lung adenocarcinoma; AD, adenocarcinoma; SQC, squamous cell carcinoma; Normal, normal tissue [file 12931_2023_2647_MOESM8_ESM.jpg]

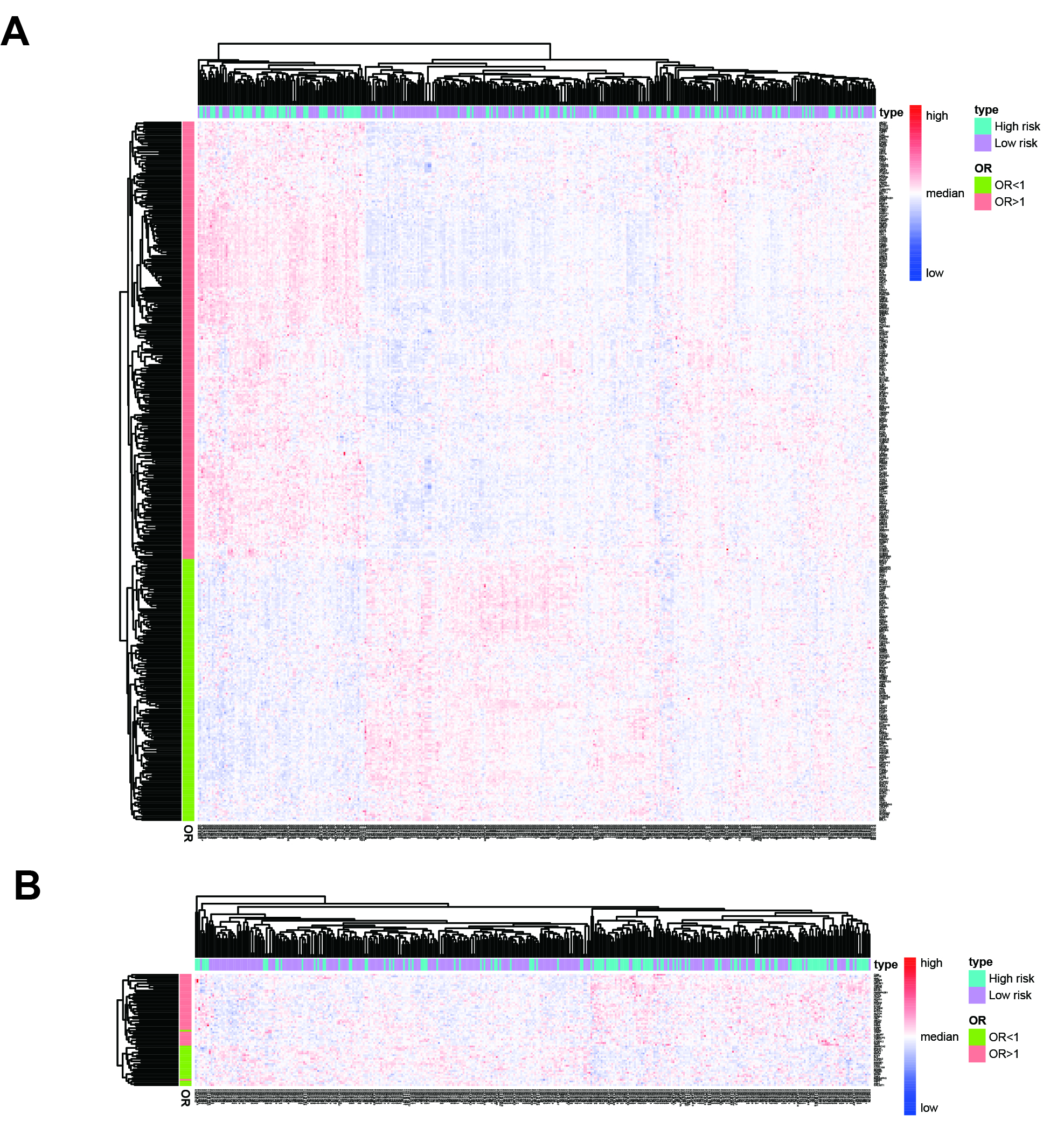

Supplement: Supplementary file 9 — Additional file 9: Figure S2. Heatmaps for 398 risk-related genes and 64 LASSO screened genes. Unsupervised heatmaps for 398 risk-related genes (A) and 64 LASSO screened genes (B); Unsupervised heatmaps where smoking related residual values were removed in the gene expression matrix for 398 risk-related genes (C) and 64 LASSO screened genes (D). OR, Odds Ratio; LASSO, least absolute shrinkage and selection operator [file 12931_2023_2647_MOESM9_ESM.zip › e-Figure 2-1.jpg]

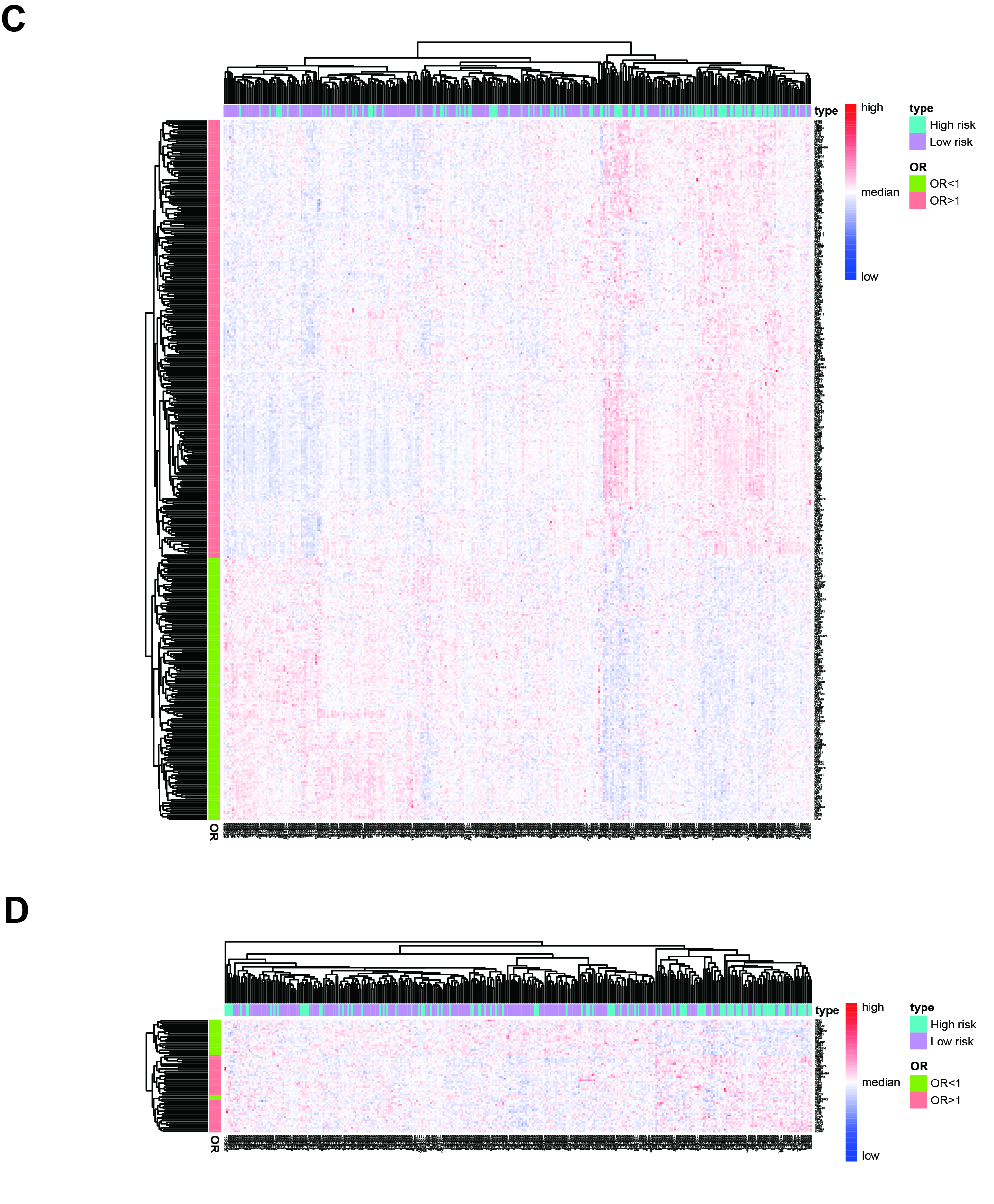

Supplement: Supplementary file 9 — Additional file 9: Figure S2. Heatmaps for 398 risk-related genes and 64 LASSO screened genes. Unsupervised heatmaps for 398 risk-related genes (A) and 64 LASSO screened genes (B); Unsupervised heatmaps where smoking related residual values were removed in the gene expression matrix for 398 risk-related genes (C) and 64 LASSO screened genes (D). OR, Odds Ratio; LASSO, least absolute shrinkage and selection operator [file 12931_2023_2647_MOESM9_ESM.zip › e-Figure 2-2.jpg]

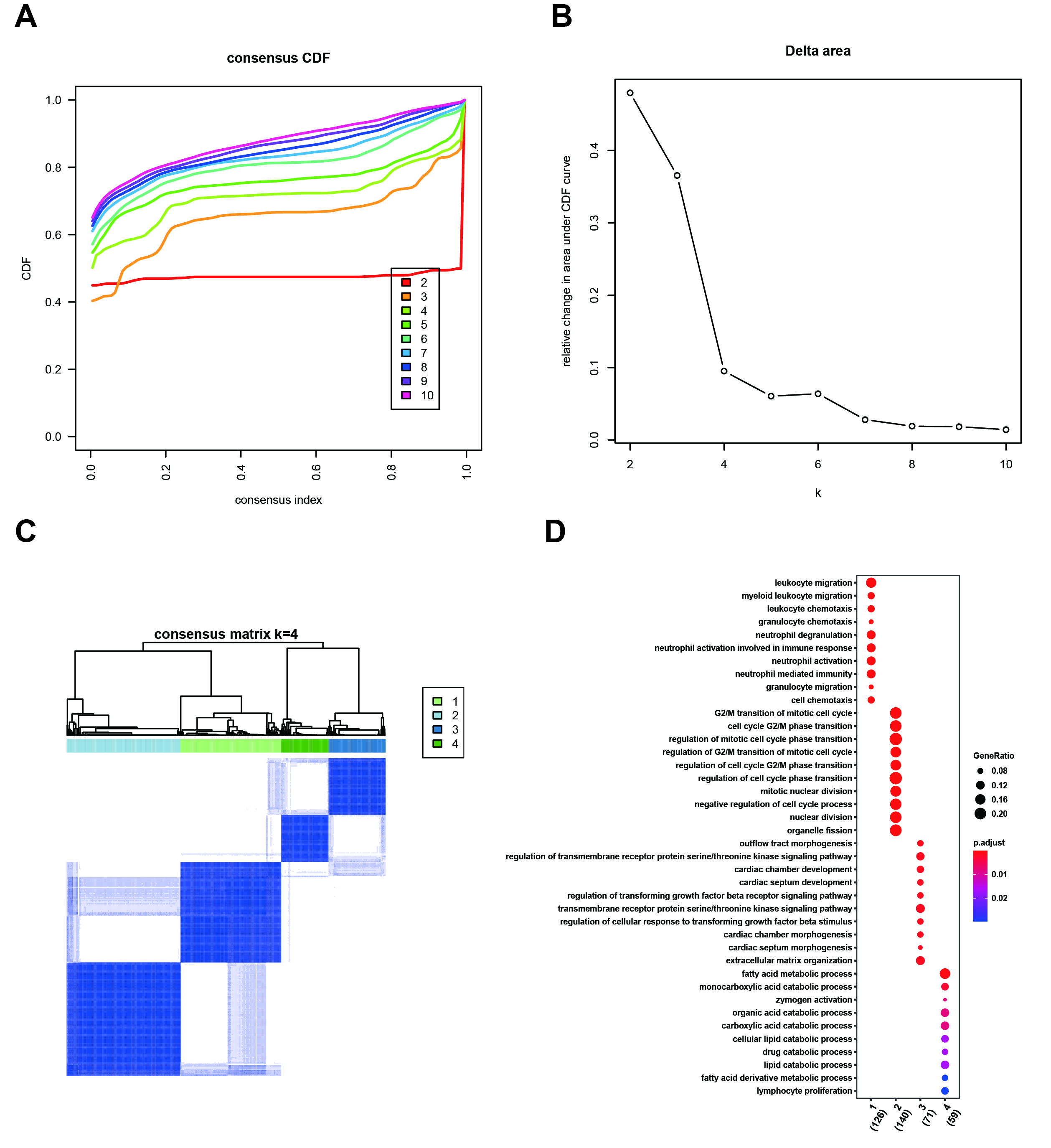

Supplement: Supplementary file 10 — Additional file 10: Figure S3. Feature selection of genes representing different patterns. Consensus cumulative distribution function (CDF) (A) and Delta area (B) of consistent cluster analysis of genes through unsupervised clustering; consensus clustering of genes into four clusters (C); GO analysis of four clusters (D); prediction effect of 114 genes into the logistic regression model (classifying high-risk, H and low-risk, L) in training set (E) and external validation set (GSE30219) (F); prediction effect of 41 genes with significant coefficients into the logistic regression model (classifying high-risk, H and low-risk, L) in training set (G) and external validation set (GSE30219) (H); Heatmaps of 114 genes in the training set (original: I, smoking related residual values removed: K) and the validation set (J) respectively; GO analysis of 64 genes of IA score (L); Expression distribution of risk genes or oncogenes ((OR > 1) (M) and protective genes or tumor suppressor genes (OR < 1) (N) in early-stage LUAD (AIS, MIA and IAC). CDF, Consensus cumulative distribution function, OR, Odds Ratio; LASSO, least absolute shrinkage and selection operator; LUAD, lung adenocarcinoma; carcinoma in situ (AIS), microinvasive adenocarcinoma (MIA), invasive adenocarcinoma (IAC) [file 12931_2023_2647_MOESM10_ESM.zip › e-Figure 3-1.jpg]

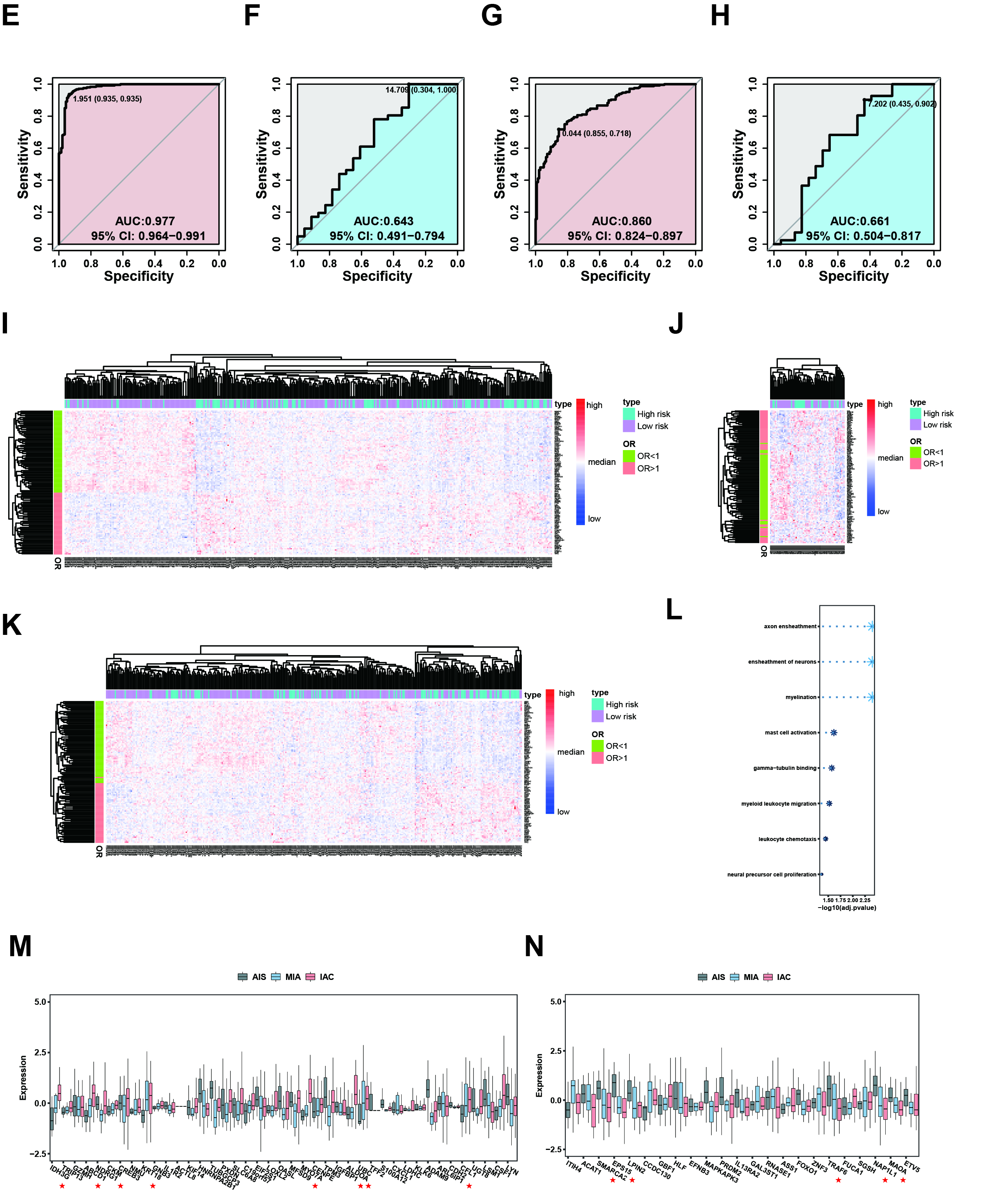

Supplement: Supplementary file 10 — Additional file 10: Figure S3. Feature selection of genes representing different patterns. Consensus cumulative distribution function (CDF) (A) and Delta area (B) of consistent cluster analysis of genes through unsupervised clustering; consensus clustering of genes into four clusters (C); GO analysis of four clusters (D); prediction effect of 114 genes into the logistic regression model (classifying high-risk, H and low-risk, L) in training set (E) and external validation set (GSE30219) (F); prediction effect of 41 genes with significant coefficients into the logistic regression model (classifying high-risk, H and low-risk, L) in training set (G) and external validation set (GSE30219) (H); Heatmaps of 114 genes in the training set (original: I, smoking related residual values removed: K) and the validation set (J) respectively; GO analysis of 64 genes of IA score (L); Expression distribution of risk genes or oncogenes ((OR > 1) (M) and protective genes or tumor suppressor genes (OR < 1) (N) in early-stage LUAD (AIS, MIA and IAC). CDF, Consensus cumulative distribution function, OR, Odds Ratio; LASSO, least absolute shrinkage and selection operator; LUAD, lung adenocarcinoma; carcinoma in situ (AIS), microinvasive adenocarcinoma (MIA), invasive adenocarcinoma (IAC) [file 12931_2023_2647_MOESM10_ESM.zip › e-Figure 3-2.jpg]

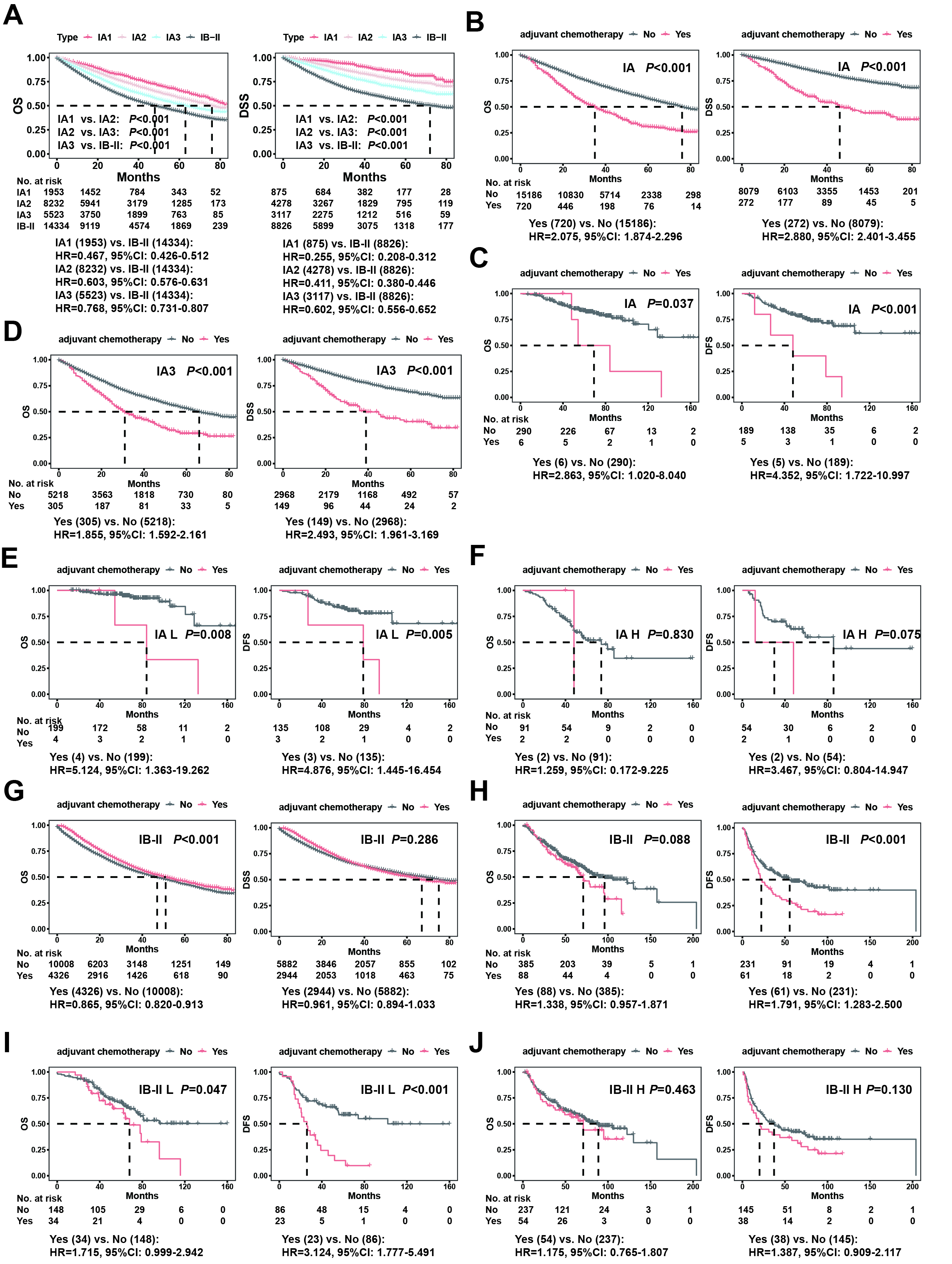

Supplement: Supplementary file 11 — Additional file 11: Figure S4. The effect of IA risk classification in the 8th TNM staging and the indication of IA score for chemotherapy sensitivity or not. (A) OS (left) and DSS (right) curves among IA1, IA2, IA3 and IB-II LUAD patients in SEER database; (B) OS (left) and DSS (right) curves between two categories of stage IA LUAD patients divided by receiving chemotherapy or not in SEER database (Some IA patients have no defined subclass); (C) OS (left) and DFS (right) curves between two categories of stage IA LUAD patients divided by receiving chemotherapy or not in GEO datasets possessing data about chemotherapy or not (GSE13213, GSE31210, GSE42127, GSE68465); (D) OS (left) and DSS (right) curves between two categories of stage IA3 LUAD patients divided by receiving chemotherapy or not in SEER database; (E) OS (left) and DFS (right) curves between two categories of stage IA L (low-risk type classified by IA score) LUAD patients divided by receiving chemotherapy or not in GEO datasets; (F) OS (left) and DFS (right) curves between two categories of stage IA H (high-risk type classified by IA score) LUAD patients divided by receiving chemotherapy or not in GEO datasets; (G) OS (left) and DSS (right) curves between two categories of stage IB-II LUAD patients divided by receiving chemotherapy or not in SEER database; (H) OS (left) and DFS (right) curves between two categories of stage IB-II LUAD patients divided by receiving chemotherapy or not in GEO datasets possessing data about chemotherapy or not; (I) OS (left) and DFS (right) curves between two categories of stage IB-II L (low-risk type classified by IA score) LUAD patients divided by receiving chemotherapy or not in GEO datasets; (J) OS (left) and DFS (right) curves between two categories of stage IB-II H (high-risk type classified by IA score) LUAD patients divided by receiving chemotherapy or not in GEO datasets. LUAD, lung adenocarcinoma; OS, overall survival; DFS, disease-free survival; DSS; disease-speci [file 12931_2023_2647_MOESM11_ESM.jpg]
