# Supplementary material for: A gene-based score for the risk stratification of stage IA lung adenocarcinoma
Source: Respir Res. 2024 Jan 4;25:18. doi: 10.1186/s12931-023-02647-4 (PMC10765678; doi:10.1186/s12931-023-02647-4)
Supplement: Supplementary file 12 — Additional file 12. Supplementary text. [file 12931_2023_2647_MOESM12_ESM.docx]

**Results**

**Machine learning to establish the risk score**

IA score= ∑Expression(Genei)*Coefficient(Genei)

IA score= CDH2*(-0.1248),+NDRG1*(-0.1844),+TUBGCP3*(-0.168),+CREB3*(-0.0818),+

CENPE*(0.0014),+CFL1*(-0.0073),+NMU*(-0.1567),+CKM*(-0.0814),+

CSF1*(-0.0836),+EFNB3*(0.1637),+EIF2S1*(-0.0559),+EPS15*(0.007),+

ETV5*(0.0235),+ABCD1*(-0.007),+ALDOA*(-0.0275),+FOXO1*(0.1675),+

ARL6IP1*(-0.0022),+FUCA1*(0.0452),+LSM1*(-0.0148),+GNB3*(-0.0921),+

CXCL1*(-0.1034),+GZMH*(-0.1628),+HLF*(-0.1214),+HNRNPA2B1*(-0.1477),+

IDH3G*(-0.0245),+IGFBP1*(-0.1019),+IL13RA2*(0.1015),+ITIH4*(0.0823),+

ACAT1*(0.0176),+KRT18*(-0.1275),+LDHC*(-0.1339),+LOXL2*(-0.0116),+

LYN*(-0.0479),+MAOA*(0.0697),+ASS1*(0.0015),+MYO7A*(-0.0229),+

NAP1L1*(0.0926),+KLK6*(-0.0485),+RNASE1*(-0.0017),+S100A12*(-0.3599),+

SGSH*(0.0914),+SLC6A8*(-0.0678),+SMARCA2*(0.1131),+TFF2*(-0.1598),+

TPM3*(-0.1652),+TRAF6*(0.2684),+UBC*(-0.2289),+UGT8*(-0.0122),+

ZNF3*(0.2155),+PRDM2*(0.1294),+PXDN*(-0.0731),+IL1R2*(-0.0581),+

MAPKAPK3*(0.1425),+C19orf57*(-0.0309), +ACTL8*(0.0362),+CCDC130*(0.03),+

MFSD9*(-0.0036),+OASL*(-0.0456),+GBF1*(0.1084),+ADAM9*(-0.1184),+

TRIP13*(0.0547),+GAL3ST1*(0.2499),+LPIN2*(0.0234),+KIF14*(0.0713)

These gene symbols and gene IDs are presented in Additional file 3: Table S3, Additional file 4: Table S4. In 386 cases in the training set, we drew an unsupervised heatmap for 398 risk-related genes and 64 LASSO-screened genes (Additional file 9: Figure S2A, B). An unsupervised heatmap was drawn in 334 patients with a clear smoking history, where smoking-related residual values were removed in the gene expression matrix (Additional file 9: Figure S2C, D).

In addition, we took into consideration genes representing different patterns for feature selection. First, we performed consensus cluster analysis of genes through unsupervised clustering. Via analysis of the consensus cumulative distribution function (CDF) (Additional file 10: Figure S3A) and delta area (Additional file 10: Figure S3B), we selected a more suitable classification of four clusters (Additional file 10: Figure S3C). Through GO analysis, we found that four clusters were enriched in immune-related biological pathways (Cluster 1), cell cycle-related biological pathways (Cluster 2), cell transmembrane signaling (Cluster 3) and substance metabolism (Cluster 4) (Additional file 10: Figure S3D). We used LASSO to screen the characteristic genes of the above gene clusters and finally selected a total of 114 genes (Cluster 1: 46, Cluster 2:16, Cluster 3:31, Cluster 4:21). These gene symbols and gene ids are presented in Additional file 5: Table S5. We evaluated the risk classification effect of these genes on stage IA LUAD. First, we included all 114 genes in the logistic regression model and found that the stratification effect of the training set was AUC: 0.977, 95% CI: 0.964-0.991, but the validation set failed to effectively stratify: AUC: 0.643, 95% CI: 0.491-0.794 (Additional file 10: Figure S3E, F). Furthermore, we carried out a logistic regression analysis of 114 genes to obtain 41 genes with significant coefficients, which were incorporated into the logistic regression model again. We found that the stratification effect of the training set was AUC: 0.860, 95% CI: 0.824-0.897, and that of the validation set was AUC: 0.661, 95% CI: 0.504-0.817 (Additional file 10: Figure S3G, H). We found that neither the training set nor the validation set worked as well as the original 64 genes.

Therefore, we chose the 64 genes to establish the IA score for risk stratification of stage IA LUAD. Heatmaps were also generated for 114 genes in the training set and the validation set (including the original gene expression matrix and a matrix for the training set with correction for the influence of smoking history; smoking history records were unavailable for all patients in the validation set) (Additional file 10: Figure S3I-K).

We carried out gene enrichment analysis on the 64 selected genes and found that these genes were mainly enriched in tumor division and proliferation, immune infiltration, chemokines and other signaling pathways (Additional file 10: Figure S3L). In fact, the above cellular behaviors and signaling pathways play an important role in the occurrence and development of tumor malignancy, which reflects the intrinsic biological significance of IA score in tumor risk stratification and suggests that early-stage LUAD (stage IA) patients could benefit from cytotoxic chemotherapy targeting division and proliferation and immunotherapy, especially in high-risk subtypes with enhancement of aggressive biological behaviors and a poorer prognosis.

To further identify key genes and target molecules (top genes) among the above 64 genes, we used our transcriptome dataset of early-stage LUAD tissues (AIS: 20, MIA: 17, IAC: 23) (GSA-Human: HRA005169) to assess the 64 genes for further verification. Risk classification of early-stage LUAD could identify valuable molecular clues regarding the tumorigenesis process by which preneoplastic tissues transform into invasive adenocarcinoma. We analyzed the expression distribution of the above 64 genes (OR>1, risk genes or oncogenes, 41; OR<1, protective genes or tumor suppressor genes, 23) in AIS-MIA-IAC. Eight of the 41 risk-related genes (OR>1) showed statistically significant increases in expression along with disease progression (p<0.05): isocitrate dehydrogenase (NAD(+)) 3 noncatalytic subunit gamma, IDH3G; aldolase, fructose-bisphosphate A, ALDOA; myosin VIIA, MYO7A; ATP binding cassette subfamily D member 1, ABCD1; ubiquitin C, UBC; keratin 18, KRT18; cofilin 1, CFL1; creatine kinase, M-type, CKM (Additional file 10: Figure S3M). Among the 23 protective genes (OR<1), 5 genes showed statistically significant decreases in expression along with disease progression (p<0.05): epidermal growth factor receptor pathway substrate 15, EPS15; nucleosome assembly protein 1 like 1, NAP1L1; monoamine oxidase A, MAOA; TNF receptor associated factor 6, TRAF6; SWI/SNF related, matrix associated, actin dependent regulator of chromatin, subfamily a, member 2, SMARCA2 (Additional file 10: Figure S3N). These genes may play an important role in the malignant progression of early-stage LUAD and are expected to become potential targets for the diagnosis and treatment of early-stage LUAD.

**Discussion**

Isocitrate dehydrogenase (NAD(+)) 3 noncatalytic subunit gamma (IDH3G) is the main constituent subunit of isocitrate dehydrogenase (IDH). IDH is an important metabolic enzyme in the tricarboxylic acid cycle, and its mutation and abnormal expression are related to the occurrence and development of various tumors, which is expected to become a target for tumor treatment and clinical characteristic evaluation [1, 2]. It has also been reported that serum IDH can be used for the diagnosis and treatment of lung cancer, mutated IDH promotes the malignant progression of lung cancer cells, and IDH inhibitors inhibit the malignant biological behavior of lung cancer cells [3-5]. Aldolase fructose-bisphosphate A (ALDOA) is an important aldolase in the process of glycolysis that is abnormally expressed in a variety of malignant tumors and predicts poor prognosis. It promotes the malignant progression of tumors by affecting metabolism and nonenzymatic functions and is a potential target for tumor diagnosis, prognostic detection and treatment [6]. In lung cancer, ALDOA overexpression is not only an indicator of poor prognosis but also promotes malignant phenotypes such as metastasis, cell stemness, and drug resistance, and inhibitors of ALDOA show antitumor effects [7-10]. Myosin VIIA (MYO7A) belongs to the myosin gene family, and its variants are associated with human Usher syndrome, which is characterized by hearing impairments and retinal degeneration [11]. MYO7A has been reported to promote melanoma proliferation, migration and lung metastasis [12]. ATP binding cassette subfamily D member 1 (ABCD1) is a member of the superfamily of ATP-binding cassette (ABC) transporters and is likely involved in the peroxisomal transport or catabolism of very long-chain fatty acids [13]. Mutations in this gene are linked to adrenoleukodystrophy [14]. ABCD1 has rarely been studied in tumors, and its expression characteristics show heterogeneity, which may be related to cell differentiation and tumor drug resistance [15]. Ubiquitin C (UBC) is a gene encoding ubiquitin. Ubiquitin and ubiquitination are important factors in the regulation of protein function and are widely involved in cell biological processes; their abnormal regulation is closely related to the occurrence and development of tumors [16]. In lung cancer, ubiquitin presents high protein expression and silences UBC to suppress growth and increase radiosensitivity [17]. Keratin 18 (KRT18), encoding type I intermediate filament protein, which is not only a specific marker of epithelial cells, plays a structural function but is also widely involved in the cell cycle, apoptosis and regulation of cancer-related signaling pathways [18]. In lung cancer, KRT18 not only plays a diagnostic and clinical role but also promotes lung cancer migration and chemotherapy resistance [19]. Cofilin 1 (CFL1) belongs to the gene family of actin depolymerization factor/cofilin (ADF/CFL), which is widely involved in cytoplasmic division, cell apoptosis, motor and other biological behaviors, and CFL1 is highly expressed in a variety of tumors and is related to tumor growth, migration and invasion [20, 21]. In lung cancer, CFL1 is a prognostic marker and is involved in malignant features such as migration and drug resistance [22, 23]. Creatine kinase, M-type (CKM) is involved in encoding creatine kinase (CK), which is an important marker of myocardial infarction, and plasma creatine kinase content in lung cancer is associated with metastasis, recurrence and survival prognosis [24]. CKM coding subunits constitute skeletal muscle CK-MM and myocardial CK-MB, but the expression of CKM in other tissues is relatively small, and there is no relationship between tissue expression and the biological behavior of lung cancer [25, 26]. The epidermal growth factor receptor pathway substrate 15 (EPS15)-encoding protein is a substrate of the epidermal growth factor receptor (EGFR) receptor, which is involved in EGFR endocytosis and secretion and affects growth factor signaling [27]. In lung cancer, EPS15 and DNA damage-regulated autophagy modulator 1 (DRAM1) or PTPN3-mediated Eps15 dephosphorylation promoted lysosomal degradation of EGFR pairs, thereby exerting tumor inhibition effects [28, 29]. Nucleosome assembly protein 1 like 1 (NAP1L1)-encoded proteins participate in nucleosome assembly and remodeling and regulate gene transcription and DNA replication [30]. In lung cancer, NAP1L1 can evaluate clinical features and malignant prognosis and promote malignant behaviors such as tumor cell proliferation and migration [31]. Monoamine oxidase A (MAOA), which encodes an enzyme involved in the metabolism of amines, is closely related to neurological disorders [32]. The expression and function of MAOA in lung cancer are controversial. In non-small cell lung cancer (NSCLC), high expression of MAOA is associated with malignant clinical features [33]. An inhibitor of MAOA repressed paclitaxel-resistant NSCLC metastasis and growth [34]. In LUAD, MAOA showed low expression, which inhibited proliferation and played a role in cancer inhibition [35]. TNF receptor-associated factor 6 (TRAF6) encodes a protein that is an important linker molecule in the tumor necrosis factor superfamily (TNFSF) and the Toll-like/interleukin-1 receptor (TLR/ILR) superfamily, which are widely involved in the regulation of cell signaling pathways and play an important role in the development of tumors [36]. In lung cancer, TRAF6 is highly expressed, which is correlated with malignant clinical features and promotes malignant proliferation [37, 38]. SWI/SNF-related, matrix-associated, actin-dependent regulator of chromatin, subfamily a, member 2 (SMARCA2) encodes a protein involved in the formation of the switch-sucrose nonfermenting (SWI/SNF) complex and is an important complex involved in gene expression regulation [39]. In lung cancer, SMARCA2-negative expression is associated with malignant clinical features and is an independent risk factor for a poor prognosis [40]. SMARCA2 can inhibit lung cancer cell vitality, and its absence contributes to the development of lung cancer [41, 42]. In addition, loss of SWI/SNF expression is associated with PD-L1-positive status and high tumor mutation burden (TMB) in lung cancer [43].

1. Fujii T, Khawaja MR, DiNardo CD et al. Targeting isocitrate dehydrogenase (IDH) in cancer. Discov Med 2016; 21: 373-380.

2. Pirozzi CJ, Yan H. The implications of IDH mutations for cancer development and therapy. Nat Rev Clin Oncol 2021; 18: 645-661.

3. Sun N, Chen Z, Tan F et al. Isocitrate dehydrogenase 1 is a novel plasma biomarker for the diagnosis of non-small cell lung cancer. Clin Cancer Res 2013; 19: 5136-5145.

4. Yan B, Hu Y, Ma T, Wang Y. IDH1 mutation promotes lung cancer cell proliferation through methylation of Fibulin-5. Open Biol 2018; 8.

5. Park S, Lee J, Lee SY. IDH-Inhibiting Small Molecule DTDQ Inhibits Migration and Invasion of A549 Human Non-Small-Cell Lung Cancer Cells via Sequential Inactivation Of ERK and P38 Signaling Pathways. Cell Biochem Biophys 2018; 76: 255-263.

6. Chang YC, Yang YC, Tien CP et al. Roles of Aldolase Family Genes in Human Cancers and Diseases. Trends Endocrinol Metab 2018; 29: 549-559.

7. Lu G, Shi W, Zhang Y. Prognostic Implications and Immune Infiltration Analysis of ALDOA in Lung Adenocarcinoma. Front Genet 2021; 12: 721021.

8. Chang YC, Chan YC, Chang WM et al. Feedback regulation of ALDOA activates the HIF-1α/MMP9 axis to promote lung cancer progression. Cancer Lett 2017; 403: 28-36.

9. Chang YC, Yang YF, Chiou J et al. Nonenzymatic function of Aldolase A downregulates miR-145 to promote the Oct4/DUSP4/TRAF4 axis and the acquisition of lung cancer stemness. Cell Death Dis 2020; 11: 195.

10. Chang YC, Chiou J, Yang YF et al. Therapeutic Targeting of Aldolase A Interactions Inhibits Lung Cancer Metastasis and Prolongs Survival. Cancer Res 2019; 79: 4754-4766.

11. Jouret G, Poirsier C, Spodenkiewicz M et al. Genetics of Usher Syndrome: New Insights From a Meta-analysis. Otol Neurotol 2019; 40: 121-129.

12. Liu Y, Wei X, Guan L et al. Unconventional myosin VIIA promotes melanoma progression. J Cell Sci 2018; 131.

13. Tawbeh A, Gondcaille C, Trompier D, Savary S. Peroxisomal ABC Transporters: An Update. Int J Mol Sci 2021; 22.

14. Turk BR, Theda C, Fatemi A, Moser AB. X-linked adrenoleukodystrophy: Pathology, pathophysiology, diagnostic testing, newborn screening and therapies. Int J Dev Neurosci 2020; 80: 52-72.

15. Hlaváč V, Souček P. Role of family D ATP-binding cassette transporters (ABCD) in cancer. Biochem Soc Trans 2015; 43: 937-942.

16. Hwang JT, Lee A, Kho C. Ubiquitin and Ubiquitin-like Proteins in Cancer, Neurodegenerative Disorders, and Heart Diseases. Int J Mol Sci 2022; 23.

17. Tang Y, Geng Y, Luo J et al. Downregulation of ubiquitin inhibits the proliferation and radioresistance of non-small cell lung cancer cells in vitro and in vivo. Sci Rep 2015; 5: 9476.

18. Menz A, Weitbrecht T, Gorbokon N et al. Diagnostic and prognostic impact of cytokeratin 18 expression in human tumors: a tissue microarray study on 11,952 tumors. Mol Med 2021; 27: 16.

19. Zhang B, Wang J, Liu W et al. Cytokeratin 18 knockdown decreases cell migration and increases chemosensitivity in non-small cell lung cancer. J Cancer Res Clin Oncol 2016; 142: 2479-2487.

20. Kanellos G, Frame MC. Cellular functions of the ADF/cofilin family at a glance. J Cell Sci 2016; 129: 3211-3218.

21. Shishkin S, Eremina L, Pashintseva N et al. Cofilin-1 and Other ADF/Cofilin Superfamily Members in Human Malignant Cells. Int J Mol Sci 2016; 18.

22. Castro MA, Dal-Pizzol F, Zdanov S et al. CFL1 expression levels as a prognostic and drug resistance marker in nonsmall cell lung cancer. Cancer 2010; 116: 3645-3655.

23. Müller CB, de Barros RL, Castro MA et al. Validation of cofilin-1 as a biomarker in non-small cell lung cancer: application of quantitative method in a retrospective cohort. J Cancer Res Clin Oncol 2011; 137: 1309-1316.

24. Liu L, He Y, Ge G et al. Lactate dehydrogenase and creatine kinase as poor prognostic factors in lung cancer: A retrospective observational study. PLoS One 2017; 12: e0182168.

25. Yan YB. Creatine kinase in cell cycle regulation and cancer. Amino Acids 2016; 48: 1775-1784.

26. Wallimann T, Hemmer W. Creatine kinase in non-muscle tissues and cells. Mol Cell Biochem 1994; 133-134: 193-220.

27. Salcini AE, Chen H, Iannolo G et al. Epidermal growth factor pathway substrate 15, Eps15. Int J Biochem Cell Biol 1999; 31: 805-809.

28. Geng J, Zhang R, Yuan X et al. DRAM1 plays a tumor suppressor role in NSCLC cells by promoting lysosomal degradation of EGFR. Cell Death Dis 2020; 11: 768.

29. Li MY, Lai PL, Chou YT et al. Protein tyrosine phosphatase PTPN3 inhibits lung cancer cell proliferation and migration by promoting EGFR endocytic degradation. Oncogene 2015; 34: 3791-3803.

30. Attia M, Rachez C, Avner P, Rogner UC. Nucleosome assembly proteins and their interacting proteins in neuronal differentiation. Arch Biochem Biophys 2013; 534: 20-26.

31. Nagashio R, Kuchitsu Y, Igawa S et al. Prognostic significance of NAP1L1 expression in patients with early lung adenocarcinoma. Biomed Res 2020; 41: 149-159.

32. Nikolac Perkovic M, Svob Strac D, Nedic Erjavec G et al. Monoamine oxidase and agitation in psychiatric patients. Prog Neuropsychopharmacol Biol Psychiatry 2016; 69: 131-146.

33. Liu F, Hu L, Ma Y et al. Increased expression of monoamine oxidase A is associated with epithelial to mesenchymal transition and clinicopathological features in non-small cell lung cancer. Oncol Lett 2018; 15: 3245-3251.

34. Yang X, Zhao D, Li Y et al. Potential monoamine oxidase A inhibitor suppressing paclitaxel-resistant non-small cell lung cancer metastasis and growth. Thorac Cancer 2020; 11: 2858-2866.

35. Huang Y, Zhao W, Ouyang X et al. Monoamine Oxidase A Inhibits Lung Adenocarcinoma Cell Proliferation by Abrogating Aerobic Glycolysis. Front Oncol 2021; 11: 645821.

36. Li J, Liu N, Tang L et al. The relationship between TRAF6 and tumors. Cancer Cell Int 2020; 20: 429.

37. Zhang XL, Dang YW, Li P et al. Expression of tumor necrosis factor receptor-associated factor 6 in lung cancer tissues. Asian Pac J Cancer Prev 2014; 15: 10591-10596.

38. Starczynowski DT, Lockwood WW, Deléhouzée S et al. TRAF6 is an amplified oncogene bridging the RAS and NF-κB pathways in human lung cancer. J Clin Invest 2011; 121: 4095-4105.

39. Mashtalir N, D'Avino AR, Michel BC et al. Modular Organization and Assembly of SWI/SNF Family Chromatin Remodeling Complexes. Cell 2018; 175: 1272-1288.e1220.

40. Sun S, Li Q, Zhang Z et al. SMARCA2 deficiency in NSCLC: a clinicopathologic and immunohistochemical analysis of a large series from a single institution. Environ Health Prev Med 2022; 27: 3.

41. Wu J, He K, Zhang Y et al. Inactivation of SMARCA2 by promoter hypermethylation drives lung cancer development. Gene 2019; 687: 193-199.

42. Marquez-Vilendrer SB, Rai SK, Gramling SJ et al. Loss of the SWI/SNF ATPase subunits BRM and BRG1 drives lung cancer development. Oncoscience 2016; 3: 322-336.

43. Naito T, Udagawa H, Umemura S et al. Non-small cell lung cancer with loss of expression of the SWI/SNF complex is associated with aggressive clinicopathological features, PD-L1-positive status, and high tumor mutation burden. Lung Cancer 2019; 138: 35-42.
